# An Integrated Method for Evaluation of Salt Tolerance in a Tall Wheatgrass Breeding Program

**DOI:** 10.3390/plants14070983

**Published:** 2025-03-22

**Authors:** Qiang Xiao, Wei Li, Pan Hu, Jianfeng Cheng, Qi Zheng, Hongwei Li, Zhensheng Li

**Affiliations:** 1 Key Laboratory of Seed Innovation, Institute of Genetics and Developmental Biology, Chinese Academy of Sciences, Beijing 100101, China; aa58654254@163.com (Q.X.); 2021720827@yangtzeu.edu.cn (W.L.); hupan@genetics.ac.cn (P.H.); qzheng@genetics.ac.cn (Q.Z.); zsli@genetics.ac.cn (Z.L.); 2 School of Agricultural Sciences, Jiangxi Agricultural University, Nanchang 330045, China; 3 College of Agriculture, Yangtze University, Jingzhou 434000, China

**Keywords:** tall wheatgrass, salt tolerance, saline–alkaline soils, gene expression

## Abstract

Tall wheatgrass, a perennial forage grass renowned for its salt–alkali tolerance, has recently been proposed as a key species for planting in coastal saline–alkaline lands to establish a “Coastal Grass Belt”. Highly salt-tolerant and high-yielding varieties are essential to achieve this objective. To enhance breeding efficiency, a method integrating seed germination, seedling emergence, and seedling growth was established to evaluate salt tolerance in tall wheatgrass. Germination tests revealed that under 250 mM NaCl, 150 mM Na_2_SO_4_, 150 mM NaHCO_3_, or 100 mM Na_2_CO_3_, the relative seed germination rates were 31.5%, 65.4%, 68.2%, and 32.6%, respectively, compared to the non-stress condition. Germination tests can use 250 mM NaCl and 100 mM Na_2_CO_3_ to assess tall wheatgrass tolerance to neutral and sodic salt stress, respectively. In addition, 250 mM NaCl or saline water with EC_w_ = 6.6 dS m^−1^ resulted in relative seedling emergence rates of 52% and 59.8%, respectively, compared to the non-stress condition. Seedling hydroponic culture demonstrated that exposure to 300 mM NaCl resulted in relative total dry weight, shoot dry weight, and root dry weight of 38.2%, 35.7% and 50%, respectively, compared to the non-stress condition. Salt-response genes exhibited differential expression in tall wheatgrass under long-term and short-term salt stress. Interestingly, the expression levels of *NHX7.1* and *NCL1* were significantly higher in salt-tolerant lines compared to salt-sensitive lines. Based on an integrated evaluation of seed germination, seedling emergence, and seedling growth, five out of the 28 tall wheatgrass lines were identified as salt-tolerant. Additionally, two *Tritipyrum* lines, derived from the cross of *Triticum aestivum* cv. Xinong 6028 and *Thinopyrum ponticum* line Zhongyan 1, were found to inherit salt tolerance from tall wheatgrass. Collectively, this work provided an integrated method for salt tolerance testing in a tall wheatgrass breeding program.

## 1. Introduction

Tall wheatgrass (*Thinopyrum ponticum* (Podp.) Z. W. Liu & R. C. Wang, 2n = 10x = 70) is a perennial cool-season bunchgrass that has been widely cultivated in America, Australia, and some European countries since the 1950s [1,2]. For instance, in Argentina there are currently 1 × 10^6^ ha of tall wheatgrass pasture in the Salado River basin [3]. Tall wheatgrass exhibits a high level of tolerance to salt and alkali [2,4,5,6], making it an ideal grass for saline pastures. To meet the ever-increasing demand for food of a growing human population, it is crucial to balance and avoid competition for arable land between food crops and forage crops. Planting salt–alkali tolerant forage crops, including tall wheatgrass, in saline–alkaline land can alleviate such competition [7,8,9,10,11]. Recently, the concept of a “Coastal Grass Belt” has been proposed, suggesting cultivation of tall wheatgrass in the coastal saline–alkaline soils around the Bohai Sea [12,13,14]. Therefore, it is essential to breed tall wheatgrass varieties with enhanced salt tolerance. Salt tolerance in plants is a complex trait determined by multiple genes and environmental factors [15]. Reliable phenotyping of salt tolerance is pivotal for selection of salt-tolerant varieties, which largely determines breeding efficiency.

Tall wheatgrass is considered one of the most salt-tolerant forage grasses, producing higher relative herbage yield than other forage crops, including barley, perennial ryegrass, tall fescue, sudangrass, bermudagrass, orchardgrass, lovegrass [1], and alfalfa [15], under salt stress. An early study showed that the most salt-tolerant tall wheatgrass accessions among the 32 investigated accessions showed a 95–100% recovery rate in a stepwise increase to a final salinity of 765 mM equivalent of NaCl and CaCl_2_ [16]. Furthermore, another study demonstrated that two of the nine tall wheatgrass accessions exhibited 87–93% survival rates under a stepwise increase in salinity, reaching a final concentration of 750 mM NaCl [17]. In addition, Roundy (1983) found that tall wheatgrass can survive when the osmotic potential declines to −35 bar but grows slowly below −10 bar under salt stress [18]. Tall wheatgrass produced 5.9–8.3 t ha^−1^ dry matter yield when grown on saline soil with electric conductivity of “saturation paste extract” (EC_e_) = 19 dS m^−1^ [19]. An early report showed that the EC of irrigation water (EC_w_) = 7.5 dS m^−1^ was the threshold salinity for tall wheatgrass without growth reduction [20]. Furthermore, Riedell (2016) demonstrated that the salinity threshold for tall wheatgrass cv. Akar was EC_w_ = 10 dS m^−1^ without shoot dry weight (SDW) loss and EC_w_ = 30.2 dS m^−1^ for 50% SDW reduction [21]. Additionally, a recent study revealed that tall wheatgrass plants irrigated with 300 mM NaCl in a 90 d growth period reduced approximately one-third forage yield [22]. Recently, it was suggested that in the “Coastal Grass Belt” targeted area, such as the Yellow River Delta Region, tall wheatgrass can be planted in saline soils with a salinity of less than 1% (*w*/*w*) [23]. Moreover, to achieve high forage productivity, irrigation with saline drainage water having a salinity of less than 5 g L^−1^ was recommended for tall wheatgrass in Spring as needed [24]. The surface soil salinity in the “Coastal Grass Belt” targeted region ranges between 0.3% and 1.0% (*w*/*w*), with the maximum salinity exceeding 3.0% [25], making it unprofitable for other food crops. Therefore, screening and selecting highly salt-tolerant tall wheatgrass varieties, rather than food crops, offers a promising approach to efficiently utilize such coastal saline soils.

Salt tolerance in tall wheatgrass has typically been assessed through various methods, including seed germination tests in the laboratory [4,26,27,28,29,30,31,32], seedling emergence [27,28], seedling growth evaluations using hydroponic culture supplemented with NaCl [17,26,33,34], and experiments in sand- or soil-filled pots irrigated with NaCl solution or saline water in greenhouses [4,16,18,21,22,27,28,35,36,37,38,39,40,41]. Additionally, field evaluations have been conducted through irrigation with saline water or NaCl solution, as well as on-side field tests in saline and/or alkaline soils [6,23,42,43,44,45]. Due to the differences of experimental conditions and plant materials, the aforementioned evaluation results of salt tolerance in tall wheatgrass were not always consistent. In addition, the testing methods developed for physiological and biochemical analysis fail to replicate real field conditions and may be unsuitable for tall wheatgrass breeding programs. Although seed germination in 220 mM NaCl solution has been suggested as a selection index for salt tolerance in an Argentinian tall wheatgrass breeding program [31], seed germination results for salt tolerance are often inconsistent with seedling tolerance under field conditions [4]. Considering that seed germination, seedling emergence, and seedling growth all determine the ultimate forage yield and persistence of tall wheatgrass on saline–alkaline soils, the evaluation of salt tolerance in tall wheatgrass breeding programs should fully consider the performance across all these three aspects. Therefore, an integrated approach that comprehensively evaluates these three aspects can provide thorough and reliable information for assessing the salt tolerance of tall wheatgrass under real field conditions for practical applications.

The salt tolerance in plants is regulated by genes, which respond differentially to salt stress. Several studies were conducted on the gene expression of diploid *T. elongata*, a possible ancestor subgenome donor of tall wheatgrass, and wheat × *T. elongata* amphiploids responding to salt stress. Gulick and Dvorák (1987) firstly found that 18 mRNA species were differentially accumulated in a wheat–*T. elongata* amphiploid [46]. Then, eleven of these mRNA species were cloned as cDNA, which are induced in *T. elongata* within 2 h and peaked at 6 h of 250 mM NaCl treatment [47]. These genes are early salt stress induced, which accumulated greater in roots than in shoots in *T. elongata* [48]. In addition, dehydrin and non-specific lipid transfer protein genes were also induced by salt in *T. elongata* [49]. Further, Shen et al. (2001) reported a salt- and ABA-induced protein kinase gene, *Esi47*, in *T. elongata* [50]. In addition, transcriptomic analysis was also carried out on a wheat × *T. elongata* amphiploid [51,52], accounting for more salt-induced differentially expressed genes. In addition, overexpression of *AeNHX1* from *T. elongata* enhanced salt tolerance in *Arabidopsis* and *Festuca* plants [53].

Recently, Sheikh-Mohamadi et al. (2022) compared six ecotypes of tall wheatgrass, native to Iran, for salt tolerance and expression of salt-response genes [54]. This study demonstrated that the salinity-resistant ecotypes exhibited high expression levels of *NHX1*, *NHX2*, *HKT1;4*, *SnRK2.4,* and *NAC9* in leaves and roots. Especially, the salt-tolerant tall wheatgrass lines showed higher expression of *HKT1;4* and *NAC9* in leaves and roots than the salt-sensitive lines. Additionally, the transgenic *Arabidopsis* overexpressing *HKT1;4* from tall wheatgrass showed significant tolerance to salt stress [55]. Collectively, the reports on the gene expression of tall wheatgrass responding to salt stress are very limited, which hinders the molecular breeding of tall wheatgrass with enhanced salt tolerance.

The objectives in this study were to (1) establish an integrated method for evaluating salt tolerance in tall wheatgrass for breeding purposes; (2) study the expression levels of salt-response genes in tall wheatgrass under salt stress; (3) assess salt tolerance of 28 tall wheatgrass lines and five *Tritipyrum* lines derived from the cross of wheat × tall wheatgrass. *Tritipyrum* (or partial amphiploids), coined from the offspring of the cross between *Triticum aestivum* (wheat) and *Thinopyrum* species, is considered a new type of forage crop [56], which was also included in this work. The salt-tolerant tall wheatgrass or *Tritipyrum* varieties can play a key role in coastal saline and alkaline lands and other marginal lands as a forage and energy crop [8,10,11].

## 2. Results

### 2.1. Seed Germination Rate of Tall Wheatgrass Responding to NaCl, Na_2_SO_4_, NaHCO_3_, and Na_2_CO_3_

**Figure 1:**
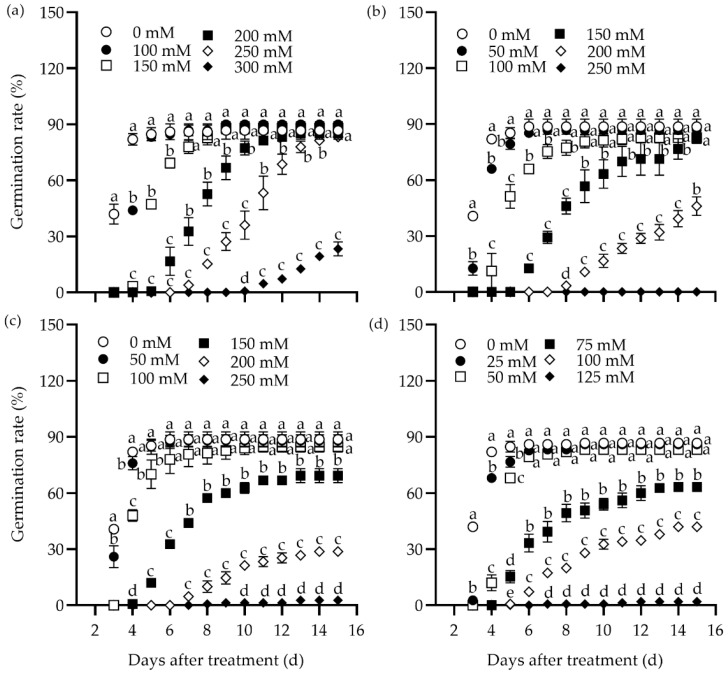
Dynamic responses of the germination rate in the tall wheatgrass line Zhongyan 1 to varying concentrations of NaCl (**a**), Na_2_SO_4_ (**b**), NaHCO_3_ (**c**), and Na_2_CO_3_ (**d**). Data are represented as mean ± SE (n = 3). Different letters indicate that the difference was significant at *p* < 0.05.

The effect of different concentrations of NaCl, Na_2_SO_4_, NaHCO_3_, and Na_2_CO_3_ on the germination rate of tall wheatgrass seeds from the line Zhongyan 1 was evaluated (Figure 1). All four salts inhibited germination rate substantially. In addition, the germination potential, germination index, vigor index, and shoot height declined drastically, while the relative salt damage rate and inhibition of shoot height elevated with rising salt concentration (Table S1). The highest concentration of salts, including 300 mM NaCl, 250 mM Na_2_SO_4_, 250 mM NaHCO_3_, and 125 mM Na_2_CO_3_, resulted in the lowest germination rates, with relative germination rates of 26.9%, 0%, 3.0%, and 2.3%, respectively, compared to the non-stress control at 15 days after treatment (DAT). It was evident that the germination rates were too low to be used for evaluating salt tolerance in tall wheatgrass. Comparatively, 250 mM NaCl, 150 mM Na_2_SO_4_, 150 mM NaHCO_3_, and 100 mM Na_2_CO_3_ resulted in relative germination rates of 31.5%, 65.4%, 68.2%, and 32.6%, respectively, and seedling height inhibition rates of 73.5%, 57.6%, 58.1%, and 73.4%, respectively, at 15 DAT. It appears that such salt stress levels may be suitable for germination tests in tall wheatgrass to evaluate salt tolerance under relatively high selection pressure. Specifically, 250 mM NaCl and 100 mM Na_2_CO_3_ can be used to assess tolerance to neutral and sodic salt stress, respectively.

### 2.2. Seedling Emergence Rate of Tall Wheatgrass Responding to NaCl and Saline Water

**Figure 2:**
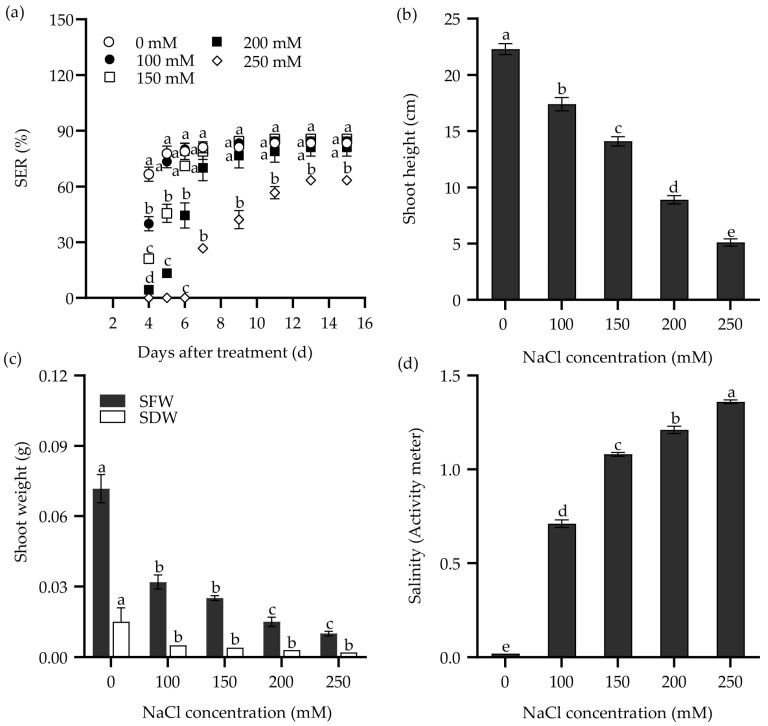
The seedling emergence rate (SER) (**a**), shoot height (**b**), and shoot weight (SFW, shoot fresh weight; SDW, shoot dry weight) (**c**) of the tall wheatgrass line Zhongyan 1 subjected to varying concentrations of NaCl solution. (**d**) The salinity of vermiculite medium after NaCl treatment, which was measured with a PNT3000 activity meter. Data are represented as mean ± SE (n = 3 for (**a**,**c**); n = 20 for (**b**,**d**)). Different letters indicate that the difference was significant at *p* < 0.05.

Furthermore, both NaCl solution and saline water were used to study the response of the seedling emergence rate (SER) in Zhongyan 1 to salt stress. As shown in Figure 2, the SER, shoot height, shoot fresh weight (SFW), and SDW declined drastically with increasing NaCl concentration. The SER reached the highest value at 11 DAT under 100 and 150 mM NaCl, which was two days later than the same NaCl treatments in the seed germination test. Meanwhile, it reached the highest value under 200 and 250 mM NaCl at 13 DAT, consistent with the same NaCl treatments in the seed germination test. When exposed to 250 mM NaCl for 9 d, the SER of Zhongyan 1 was 52% of the non-stress control. At 15 DAT, Zhongyan 1 treated with 250 mM NaCl exhibited inhibition rates of 24%, 77.2%, 86.1%, and 86.8% for SER, shoot height, SFW, and SDW, respectively, compared to the non-stress control.

**Figure 3:**
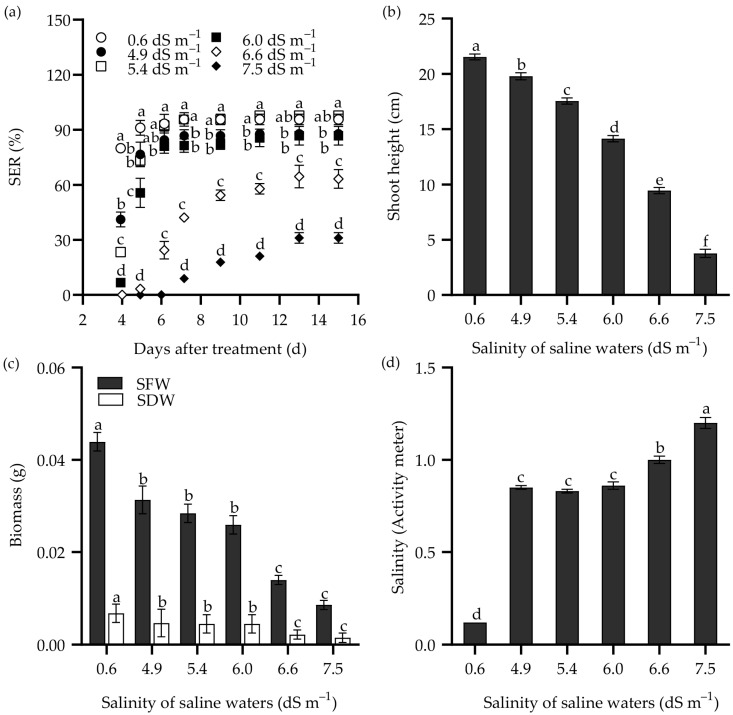
The seedling emergence rate (SER) (**a**), shoot height (**b**), and shoot weight (SFW, shoot fresh weight; SDW, shoot dry weight) (**c**) of the tall wheatgrass line Zhongyan 1 subjected to saline water. (**d**) The salinity of cultured vermiculite medium after saline water treatment, which was measured with a PNT3000 activity meter. Data are represented as mean ± SE (n = 3 for (**a**,**c**); n = 20 for (**b**,**d**)). Different letters indicate that the difference was significant at *p* < 0.05.

The response of SER in Zhongyan 1 to saline water is illustrated in Figure 3. Consistently, the SER, shoot height, SFW, and SDW decreased with increasing salinity in the saline waters. The SER reached its highest values at 11 DAT in saline waters having salinity of EC_w_ = 4.9 and 5.4 dS m^−1^, which was similar to the effects of 100 and 150 mM NaCl. Meanwhile, it reached the highest values at 13 DAT when the salinity of saline water increased to 6.0, 6.6, and 7.5 dS m^−1^, consistent with the effects of 200 and 250 mM NaCl. Under the treatment with salinity of EC_w_ = 6.6 dS m^−1^ at 9 DAT, the SER was 59.8% of the non-stress control. By 15 DAT, this treatment resulted in inhibition rates of 33.7%, 56.1%, 68.1%, and 67.6% for SER, shoot height, SFW, and SDW, respectively, compared to the non-stress control. Both NaCl and saline water treatment increased the salinity in the culture medium (Figure 2d and Figure 3d). It seems that the treatment with 250 mM NaCl or saline water with EC_w_ = 6.6 dS m^−1^ for 9 d may be suitable for evaluating SER in tall wheatgrass under salt stress.

### 2.3. Seedling Growth of Tall Wheatgrass Responding to NaCl Stress

**Figure 4:**
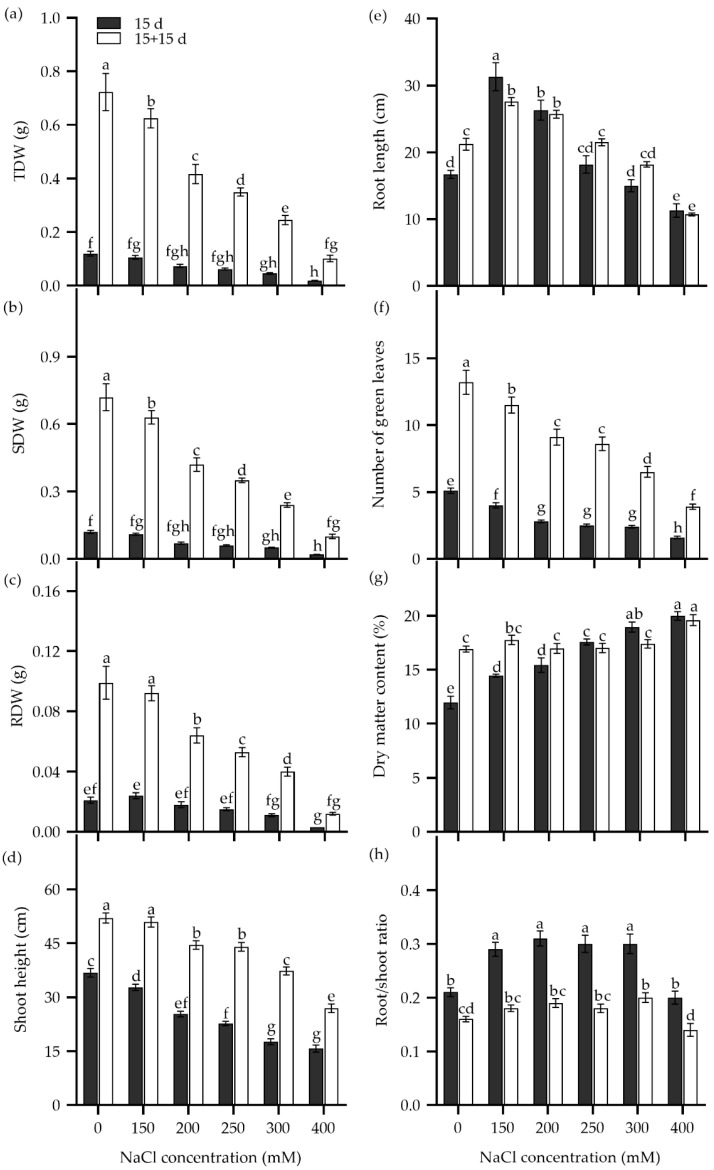
The total dry weight (TDW) (**a**), shoot dry weight (SDW) (**b**), root dry weight (RDW) (**c**), shoot height (**d**), root length (**e**), number of green leaves (**f**), dry matter content (**g**), and ratio of root to shoot dry weight (root/shoot ratio) (**h**) in the tall wheatgrass line Zhongyan 1 subjected to NaCl stress for 15 d (dark) and recovery for another 15 d after removal of NaCl (white). Data are represented as mean ± SE (n = 20). Different letters indicate that the difference was significant at *p* < 0.05.

The survival rate of Zhongyan 1 declined drastically when the NaCl concentration in the hydroponic nutrient medium was increased from 400 to 800 mM (Figure S1). Therefore, seedling growth traits were analyzed in seedlings subjected to 150–400 mM NaCl stress (Figure 4). After being cultured in nutrient solution supplemented with more than 200 mM NaCl for 15 d, the total dry matter weight (TDW), SDW, and root dry weight (RDW) decreased significantly compared to the non-stress control (Figure 4a–c). For instance, treatments with 200, 250, 300, and 400 mM NaCl resulted in inhibition rates of 38.7%, 48.7%, 61.8%, and 84.1%, respectively, for TDW compared to the non-stress control. In addition, shoot height and the number of green leaves decreased, while dry matter content and the number of senescent leaves elevated with rising NaCl concentration. Root length increased significantly under 150 and 200 mM NaCl treatments but decreased significantly in the 400 mM NaCl solution compared to the non-stress control. The ratio of root to shoot dry weight (root/shoot) increased significantly under 150–300 mM NaCl stress but returned to the non-stress level at 400 mM NaCl.

To explore the recovery of seedling growth, NaCl was removed, and the Zhongyan 1 seedlings were cultured in nutrient solution for an additional 15 d. As shown in Figure 4, the TDW, SDW, RDW, shoot height, and number of green leaves increased, while the root/shoot ratio declined. The increments in dry matter and the number of green leaves declined with the pervious increase in NaCl concentration. For instance, after removal of salt stress and recovery for 15 d, the growth inhibition rates for the 150, 200, 250, 300, and 400 mM NaCl treatments were 13.5%, 42.3%, 51.6%, 66.2%, and 85.9% of TDW under non-stress, respectively. Comparatively, the dry matter content of seedlings increased significantly under 150 and 200 mM NaCl but decreased slightly under 250–400 mM NaCl after the removal of NaCl stress. Taken together, a 15 d treatment with 300 mM NaCl resulted in inhibition rates of 50%, 64.3%, and 61.8% for RDW, SDW, and TDW, respectively, which may be suitable for evaluation of seedling tolerance to salt stress in tall wheatgrass.

### 2.4. The Effects of Seedling Age and Growth Light Intensity on Evaluation of Salt Tolerance in Tall Wheatgrass

**Figure 5:**
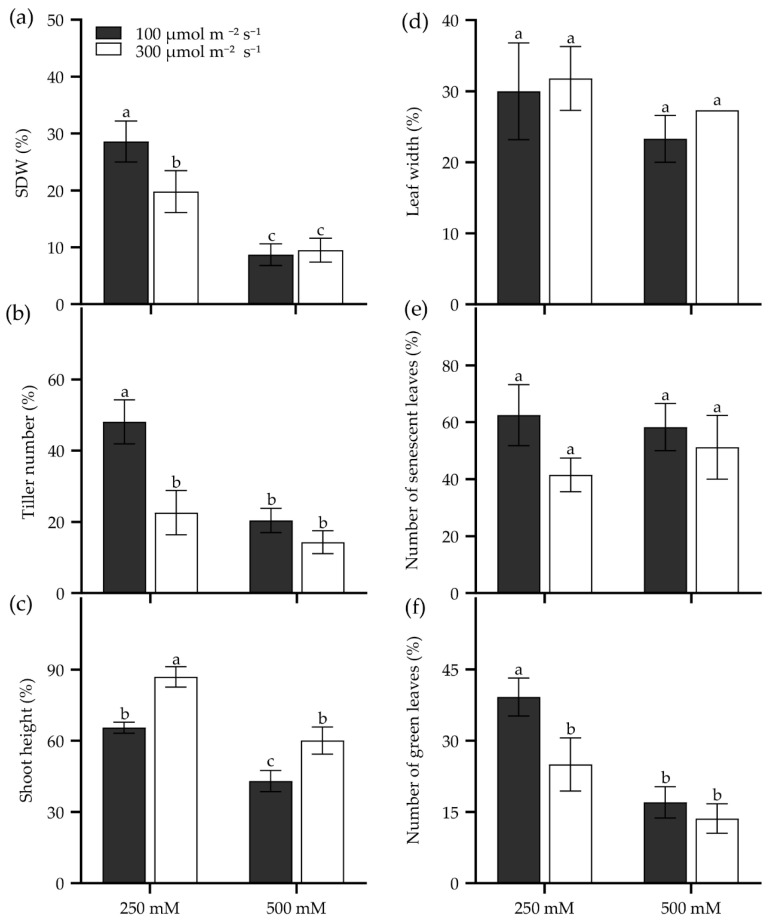
Seedling growth of the tall wheatgrass line Zhongyan 1 cultured in two growth light intensities subjected to NaCl stress, relative to non-stress condition. (**a**) Shoot dry weight (SDW); (**b**) tiller number; (**c**) shoot height; (**e**) leaf width; (**d**) the number of senescent leaves; (**f**) the number of green leaves. Data are represented as mean ± SE (n = 6). Different letters indicate that the difference was significant at *p* < 0.05.

Seedling age significantly influences salt tolerance in tall wheatgrass. For instance, the 7 d seedlings of Zhongyan 1 were more sensitive to 400 mM NaCl compared to 14 d, 21 d, and 28 d seedlings for its lowest TDW and the highest inhibition of dry matter yield (Figure S2). Also, growth light intensity affects seedling salt tolerance in tall wheatgrass. Two growth light intensities, 100 and 300 µmol m^−2^ s^−1^, were performed on Zhongyan 1 treated with 250 and 500 mM NaCl. The result showed that the 300 µmol m^−2^ s^−1^ of growth light intensity reduced relative SDW, tiller number, and the number of green leaves but enhanced relative shoot height in Zhongyan 1 treated with 250 mM NaCl compared to those grown under 100 µmol m^−2^ s^−1^ of light intensity (Figure 5). However, no significant differences were observed between these two light intensities for the investigated traits under 500 mM NaCl stress, which generated more severe salt damage than 250 mM NaCl (Figure 5). High light exacerbates salt stress-induced photoinhibition and impairs photosystem II repair [57], ultimately slowing down the seedling growth of tall wheatgrass.

### 2.5. Differential Expression of Salt-Response Genes in Tall Wheatgrass Seedlings Under Salt Stress

**Figure 6:**
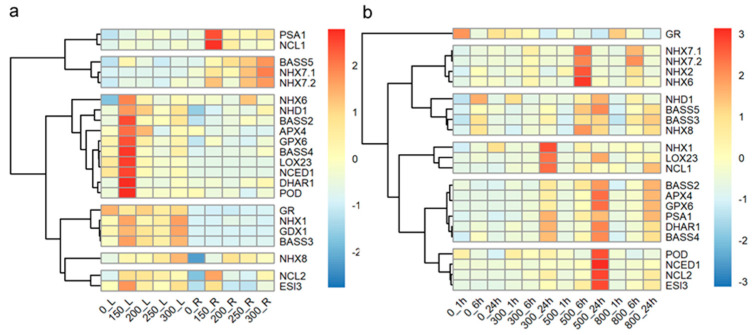
The hierarchy cluster analysis of the 22 salt-response genes in fully expanded leaf and root of the tall wheatgrass line Zhongyan 1 exposed to 150, 200, 250, and 300 mM NaCl for 15 d (**a**) and in leaf of Zhongyan 1 exposed to 300, 500, and 800 mM NaCl for 1 h, 6 h, and 24 h (**b**). _L indicates leaves; _R indicates roots.

To investigate the response of tall wheatgrass to long-term salt stress at the mRNA level, the expression levels of salt-response genes were assayed in the leaves and roots of Zhongyan 1 subjected to 150, 200, 250, and 300 mM NaCl stress for 15 d. As illustrated in Figure 6a, 10 of the 22 genes were highly induced in leaves by 150 mM NaCl, including *NHX6*, *NHD1*, *BASS2*, *APX4*, *GPX6*, *BASS4*, *LOX23*, *NCED1*, *DHAR1*, *POD*, and *ESI3*. Similarly, three genes, *PSA1*, *NCL1*, and *NCL2*, were highly induced in roots by 150 mM NaCl. However, three genes, *BSSS5*, *NHX7.1*, and *NHX7.2*, were all highly induced in roots by 300 mM NaCl. The transcripts of four genes, including *GR*, *NHX1*, *GPX1*, and *BASS3*, were higher in leaves than in roots, with three of these (*NHX1*, *GPX1*, and *BASS3*) being induced in leaves by 150 and 300 mM NaCl, respectively. Additionally, the expression of *NHX8* was induced in leaves by 300 mM NaCl and in roots by 200 mM NaCl.

Short-term salt stress (300, 500, and 800 mM NaCl within 24 h) affected the expression of all the investigated genes in the leaves of Zhongyan 1 (Figure 6b). Three genes, *NHX1*, *LOX23*, and *NCL1*, were highly induced after 24 h of treatment with 300 mM NaCl. Five *NHX* genes, *NHX7.1*, *NHX7.2*, *NHX2*, *NHX6*, and *NHX8*, were induced after 6 h of exposure to 500 mM NaCl, while eleven genes, including *NHD1*, *BASS5*, *BASS2*, *APX4*, *GPX6*, *DHAR1*, *BASS4*, *POD*, *NCED1*, *NCL2*, and *ESI3*, were induced after 24 h of exposure to 500 mM NaCl. The *GR* gene appeared to be inhibited, whereas *PSA1* was induced by 300, 500, and 800 mM NaCl treatments for 24 h. The *BASS3* gene was induced by 500 and 800 mM NaCl for 6 and 24 h, respectively. Overall, both long- or short-term salt stress, resulting from different concentrations of NaCl, led to differential expression of salt-response genes. Specifically, the expression of salt-response genes in leaves was predominantly induced by long term (15 d) treatment with 150 mM NaCl and by short-term exposure to 500 mM NaCl.

### 2.6. Expression of Salt-Response Genes in Salt-Tolerant and Salt-Sensitive Tall Wheatgrass Lines

**Figure 7:**
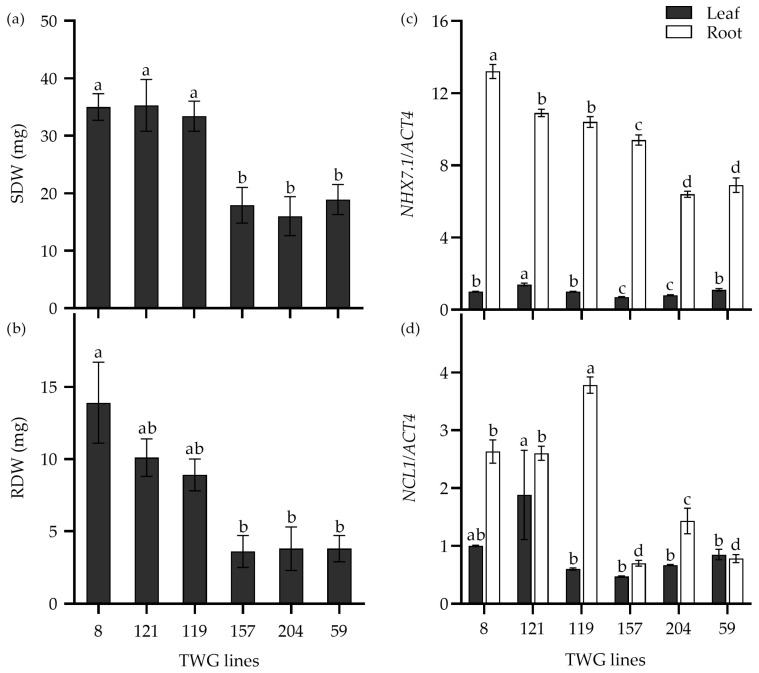
The shoot dry weight (SDW) (**a**), root dry weight (RDW) (**b**), and relative expression of *NHX7.1* (**c**) and *NCL1* (**d**) in six tall wheatgrass lines exposed to 300 mM NaCl stress for 15 d. Data are represented as mean ± SE (n = 8 for (**a**,**b**); n = 4 for (**c**,**d**)). Different letters indicate that the difference was significant at *p* < 0.05. The actin gene *ACT4* was taken as an internal reference gene.

Furthermore, the expression levels of the above salt-response genes were analyzed in six tall wheatgrass lines that exhibited contrasting seedling growth in response to 300 mM NaCl stress. For instance, the SDW and RDW of the salt-tolerant lines TWG 8, 119, and 121 were significantly higher than the salt-sensitive lines TWG 59, 157, and 204 when subjected to 300 mM NaCl stress for 15 d (Figure 7a,b). Interestingly, the expression levels of two genes, *NHX7.1* and *NCL1*, in roots were significantly higher in the salt-tolerant lines than in the salt-sensitive lines, indicating that the expression levels of these two genes appeared to be associated with the salt tolerance of the salt-tolerant lines.

### 2.7. Evaluation of Salt Tolerance in 28 Tall Wheatgrass Lines

Seed germination, seedling emergence, and seedling growth traits were used to evaluate salinity tolerance in 28 different tall wheatgrass lines compared to the control (Table 1). According to the hierarchical cluster analysis, the 28 TWG lines were classified as six groups (Figure S3). Each of the first three groups consisted of more than three lines, which was further analyzed. One-way ANOVA analysis revealed that Group 1 exhibited a significantly higher relative germination rate, germination index, shoot height, and vigor index but a lower relative salt damage rate compared to Group 3 under both 250 mM NaCl and 100 mM NaCO_3_ stress. In addition, Group 1 had a significantly higher relative SER under 250 mM NaCl and a greater SFW, TFW, SDW, and TDW under 300 mM NaCl than Group 3. Group 2 displayed intermediate values for these investigated traits. Based on these findings, Group 1, consisting of five lines, TWG8, TWG22, TWG86, TWG216, and TWG223, was the most salt-tolerant group, which may make it the superior choice to be used in the “Coastal Grass Belt” targeted region.

### 2.8. Evaluation of Salt Tolerance in Five Tritipyrum Lines Derived from Wheat × Tall Wheatgrass

**Figure 8:**
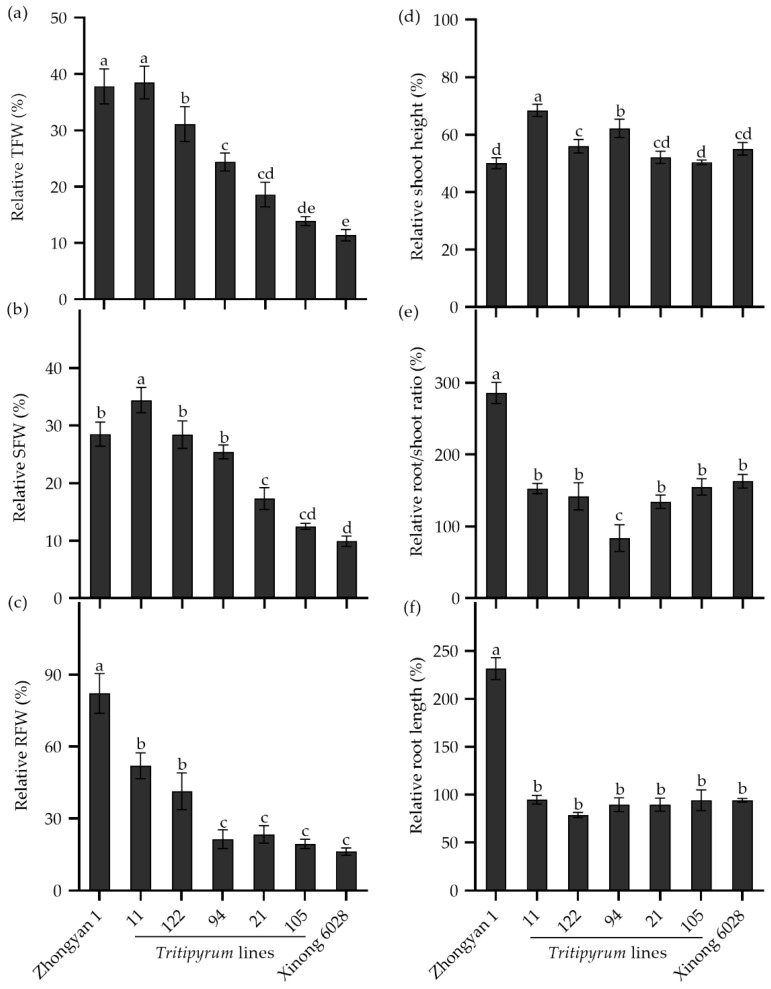
The relative seedling growth traits in the *Tritipyrum* lines and their parents Zhongyan 1 and Xinong 6028 under salt stress relative to non-stress control. (**a**) Relative TFW, relative total fresh weight; (**b**) relative SFW, relative shoot fresh weight; (**c**) relative RFW, relative root fresh weight; (**d**) relative shoot height; (**e**) relative ratio of root to shoot fresh weight; (**f**) relative root length. Data are represented as mean ± SE (n = 6). Different letters indicate that the difference was significant at *p* < 0.05.

To study the transference of salt tolerance from tall wheatgrass to wheat, the seedlings of five *Tritipyrum* lines, derived from the cross of wheat cv. Xinong 6028 × Zhongyan 1, were evaluated in a nutrient solution supplemented with 250 mM NaCl for 15 d. As shown in Figure 8a, the male parent Zhongyan 1 had the highest relative TFW, while the female wheat parent Xinong 6028 had the lowest. Line 11 had a comparable relative TFW to Zhongyan 1, which was significantly higher than the other *Tritipyrum* lines and Xinong 6028, followed by Line 122. Interestingly, Line 11 had the highest relative SFW, while Line 122 and Line 94 had comparable relative SFWs to Zhongyan 1 (Figure 8b). In addition, the relative RFWs in Line 11 and Line 122 were significantly lower than Zhongyan 1 but were higher than the other lines (Figure 8c). The relative shoot heights in Line 11, Line 122, and Line 94 were significantly higher than those in parents (Figure 8d). The relative root/shoot ratio and relative root length in the offspring and Xinong 6028 was significantly lower than Zhongyan 1 (Figure 8e,f). Collectively, Line 11 and Line 122 seemed to inherit salt tolerance from Zhongyan 1, which may be used as a salt-tolerant forage crop in the “Coastal Grass Belt” targeted area.

## 3. Discussion

Although tall wheatgrass presents a high level of salt tolerance [2], highly salt-tolerant varieties are essential for the “Coastal Grass Belt” targeted area, where the surface soil salinity is too high for profitable cultivation of food crops. Several studies have demonstrated significant genetic variation among tall wheatgrass accessions or ecotypes from different geographical locations [4,16,17,31,38,40,45], which theoretically supports the genetic improvement of tall wheatgrass for enhanced salt tolerance.

Under field conditions, any aspect of seed germination, seedling emergence, or seedling growth that is influenced by soil salinity, moisture, nutrients, and other environmental hints determines the stand establishment, final forage yield, and persistence of a tall wheatgrass pasture. First, the survival rate during seed germination and seedling emergence under salt stress determines the stand establishment of tall wheatgrass. Subsequently, the growth and regrowth performance under extreme salt stress throughout the year influences final herbage yield and persistence. Therefore, the evaluation of salt tolerance in tall wheatgrass should fully consider all aspects of seed germination, seedling emergence, and seedling growth. An ideal salt-tolerant variety should have high level of tolerance to salt stress at seed germination, seedling emergence, and seedling growth stages, ultimately producing high herbage yield on saline–alkaline soils.

### 3.1. Seed Germination Rate Under Salt Stress as a Salt Tolerance Index in Tall Wheatgrass

Seed germination tests in the laboratory are the simplest method to evaluate salt tolerance in tall wheatgrass. However, the evaluation results are not always consistent, due to variations in experimental conditions or plant materials. For instance, an early study reported that the relative germination rates of three tall wheatgrass accessions ranged from 65.6% to 80.3% under a mixed-salt solution consisting of 55 mM NaCl and 133.5 mM CaCl_2_ for 15 d [4]. Peng et al. (2002) reported that the relative germination rate of tall wheatgrass exposed to 205 mM NaCl for 9 d was approximately 22% [27]. Zhang et al. (2005) found that 50% of the non-stress control germination rate of two tall wheatgrass varieties, Tyrrell and Dundas, was achieved at approximately 300 mM NaCl. The germination rate of Tyrrel in 461.5 mM NaCl for 18 d was 8% [28]. A recent study showed that 400 mM NaCl for 11 d completely inhibited germination [32]. However, Shen et al. (1999) showed that the relative germination rate of tall wheatgrass subjected to 445 mM NaCl for 15 d was 80% [26]. In this work, 300 mM NaCl for 9 d resulted in no germination and led to decreases in germination rate, germination index, shoot height, and vigor index to 26.9%, 7.3%, 9.0%, and 0.6% of the non-stress control at 15 DAT, respectively. These conditions seemed to be too severe for testing salt tolerance in tall wheatgrass. Comparatively, 250 mM NaCl for 9 d may be more suitable for salt tolerance assessment of tall wheatgrass, considering the shorter evaluation period and relatively high selection pressure, which was consistent with the previous study [31].

Besides NaCl, other salts have also been used to test sat tolerance of tall wheatgrass. For instance, Liu et al. (2007) showed that the relative germination rates of tall wheatgrass in 274 mM NaCl, 113 mM Na_2_SO_4_, 143 mM NaHCO_3_, and 75 mM Na_2_CO_3_ for 21 d were 3.6%, 54.5%, 13.9%, and 6.7% [30], respectively. In another study, Huang and Liang (2007) found that the relative germination rates of tall wheatgrass in 250 mM NaCl, 100 mM NaHCO_3_, and 100 mM Na_2_CO_3_ for 7 d were 80%, 69%, and 14% [29], respectively. Recently, Xu et al. (2020) observed that the relative germination rates of tall wheatgrass in 200 mM NaCl, 200 mM Na_2_SO_4_, 150 mM NaHCO_3_, and 100 mM Na_2_CO_3_ for 11 d were 76.6%, 62.2%, 42.3%, and 11.9% [32], respectively. They further found that 400 mM NaCl, 600 mM Na_2_SO_4_, and 150 mM Na_2_CO_3_ resulted in no germination. In our work, the treatments with 250 mM NaCl, 150 mM Na_2_SO_4_, 150 mM NaHCO_3_, or 100 mM Na_2_CO_3_ for 9 d resulted in 31.5%, 65.4%, 68.2%, and 32.6% of non-stress seed germination rate and 73.5%, 57.6%, 58.1%, and 73.4% inhibition of seedling height at 15 DAT. Such conditions may be used as selection indices to breed salt–alkali-tolerant tall wheatgrass varieties. In particular, 250 mM NaCl and 100 mM Na_2_CO_3_ can be used for germination tests of tall wheatgrass for tolerance to neutral salt stress and sodic salt stress, respectively. The 250 mM NaCl solution is equivalent to 1.46% salinity (*w*/*w*), while 100 mM Na_2_CO_3_ means 1.06% salinity and pH 11.6 under real field conditions. Such salinity is high and causes drastic inhibition of germination and seedling emergence. Usually, combining with other stresses such as soil water deficiency, the salinity threshold for cultivation of tall wheatgrass on the coastal saline and alkaline soils was considered 1.0% (*w*/*w*) [23].

### 3.2. Seedling Emergence Rate Under Salt Stress as Salt Tolerance Index in Tall Wheatgrass

The SER of tall wheatgrass declined with increasing soil salinity [27,28]. Previous studies reported that the salinity threshold for 50% of the non-stress control SER was approximately 100 mM NaCl [27,28]. In this work, the treatment with 250 mM NaCl resulted in 52% of the non-stress control SER. The salinity threshold in this work was higher than the previous studies [27,28]. Furthermore, the SER in this study was higher than the germination rate (31.5% of the non-stress) observed in the 250 mM NaCl treatment for 9 d. This difference may be attributed to the good permeability and sufficient nutrients available in the culture medium of vermiculite. In addition, saline water with EC_w_ = 6.6 dS m^−1^ led to 59.8% of non-stress SER, indicating that local saline drainage water, rather than NaCl alone, may be convenient and effective for salt tolerance testing in tall wheatgrass breeding programs.

### 3.3. Seedling Growth Under Salt Stress as Salt Tolerance Index in Tall Wheatgrass

Following seed germination tests, seedling growth is typically evaluated to assess salt tolerance in tall wheatgrass. Both stepwise increase [4,16,17,18,27] and sudden salt stress [28,34,38] were carried out to evaluate seedling growth as a salt tolerance index in tall wheatgrass. While a stepwise increase of salinity can mitigate osmotic shock stress, low concentration of NaCl increases the salt tolerance of tall wheatgrass. For instance, 100 mM and 150 mM NaCl slightly increased the seed germination rate compared to non-stress conditions. In addition, exposure to 150 mM NaCl for 15 d enhanced root weight and root length by 13% and 87%, respectively. In order to explore the effects of stepwise increase salt stress on the salt-tolerance evaluation of tall wheatgrass, this study compared the seedling growth of Zhongyan 1 under both stepwise increase and sudden salt stress (300 mM NaCl). The result showed that the stepwise increase-induced salt stress resulted in a significantly higher SDW, RDW, TDW, root/shoot ratio, shoot height, root length, tiller number, and number of green leaves compared to sudden salt stress (Figure S4). Therefore, it appeared that stepwise increase-induced salt stress led to higher salt tolerance performance, which may partially account for the inconsistency of salt tolerance in tall wheatgrass. Field conditions are more complex than laboratory testing conditions, as the soil salinity varies with precipitation, soil moisture, and seasonal changes. When tall wheatgrass is planted, the local soil salinity is relatively stable, and sudden salt stress usually occurs. Thus, sudden salt stress may better simulate field conditions than stepwise increase-induced salt stress for breeding purposes.

The results demonstrated that NaCl concentrations exceeding 500 mM drastically reduced the survival rate. Further analysis indicated that 300 mM NaCl may be a suitable concentration for evaluating seedling growth of tall wheatgrass under salt stress, as it resulted in relative TDW, SDW, and RDW of 38.2%, 35.7%, and 50%, respectively, compared to the non-stress conditions. Even after the removal of salt stress, the TDW, SDW, and RDW of Zhongyan 1 exposed to 300 mM NaCl remained at 33.8%, 32.8%, and 40.1% of non-stress control, indicating slow recovery from salt stress. A solution of 300 mM NaCl is equivalent to 1.78% salinity under field conditions. In the field, such soil salinity, combined with other environmental stresses, often results in bare land.

### 3.4. Differential Expression of Salt-Response Genes in Tall Wheatgrass Under Salt Stress

There are very few reports on gene expression in tall wheatgrass under salt stress, likely due to a lack of gene-sequence information. Only one recent study reported the expression of *NHX1*, *NHX2*, *HKT1;4*, *SnRK2.4*, and *NAC9* in six tall wheatgrass ecotypes from Iran [54]. The *NHX* genes encode Na^+^/H^+^ antiporters, which play a major role in Na^+^ exclusion by transporting it from the cytoplasm into vacuoles or extracellular spaces [58]. The *HKT* genes encode Na^+^/K^+^ transporters, which protect leaves from Na^+^ over-accumulation [59]. Recently, a tall wheatgrass *HKT1;4* gene was found to enhance salt tolerance in *Arabidopsis* [55]. The *SnRK2* genes encode non-fermenting related protein kinases, which are involved in stress-signaling responses [60]. Overexpression of *NAC* transcription factor genes has been shown to enhance salt tolerance in plants [61,62,63]. Furthermore, Sheikh-Mohamadi et al. (2022) found that the induction of *SnRK2.4* and *NAC9* was more pronounced in salt-tolerant tall wheatgrass ecotypes [54].

In this study, the expression of six *NHX* genes, four *BASS* genes encoding sodium/metabolite cotransporters, two *NCL* genes encoding sodium/calcium exchangers, antioxidant enzyme genes, and other salt-response genes was assayed in Zhongyan 1 under NaCl stress. Both long-term (15 d) and short-term (within 24 h) salt stress induced differential expression of the investigated genes. Among the ten long-term salt stress-induced genes in leaves by 150 mM NaCl, nine were also induced under short-term salt stress in leaves. For instance, five genes, including *NCED1*, *BASS2*, *APX4*, *GPX6*, and *POD*, were drastically induced by 500 mM NaCl for 24 h, two genes, *DHAR* and *BASS4*, were highly induced by both 300 and 500 mM NaCl for 24 h, and *NHX6* and *LOX23* were highly induced by 500 mM NaCl for 6 h and by 300 mM NaCl for 24 h. Under short-term stress, more genes were induced at 24 h than at 6 h, which was inconsistent with the expression of early salt-induced (*ESI*) genes (peaked at 6 h) [47]. The expression of two salt-response genes, *NHX7.1* and *NCL1*, positively correlated with seedling height, root length, TDW, SDW, and RDW in the salt-tolerant lines TWG8, TWG121, and TWG119. The *NHX7* gene, also known as *SOS1*, encodes a sodium/hydrogen antiporter; its expression level has been significantly associated with salt tolerance in wild rice species [64]. In addition, overexpression of *SOS1* enhanced salt tolerance in tobacco [65]. A very recent study demonstrated that SOS1 tonoplast neo-localization plays a role in the extreme salinity tolerance of *Salicornia bigelovii* [66]. The *NCL1* gene, encoding a sodium/calcium exchanger-like protein, participates in the maintenance of Ca^2+^ homeostasis in *Arabidopsis*. The *NCL* mutants of *Arabidopsis* exhibited enhanced salt sensitivity [67]. The correlation of the expression levels of *NHX7* and *NCL1* and biomass in salt-tolerant tall wheatgrass lines requires further investigation in a large population.

### 3.5. Screening of Salt-Tolerant Lines of Tall Wheatgrass for Breeding Purpose

Several initiatives have been undertaken to identify salt-tolerant lines of tall wheatgrass. For instance, in combination with germination tests and field evaluation, Dewey (1960) found that three tall wheatgrass accessions (Mandan 1422, NDG-1, and P.I. 109452) exhibited greater salt tolerance than the other 22 *Agropyron* species [4]. Furthermore, Shannon (1978) reported that among the 32 tall wheatgrass lines, the seven most salt-tolerant lines had 95–100% of recovery rate, which consisted of P.I. 142012, 205279, 276399, 276709, 283164, 297871, and 315352 [16]. Additionally, Mcguire and Dvôrák (1981) demonstrated that P.I. 276399 and 315352 had survival rates of 100% and 87% under 500 mM and 750 mM NaCl [17], respectively. Based on the seedling performance of nine tall wheatgrass accessions exposed to 0.9% NaCl (154 mM) for 15 d, six accessions were deemed as salt-tolerant, which included Alkar, Platte, P.I. 276399, 308592, 535580, and 595139 [38]. In addition, one of the ten adapted tall wheatgrass populations from Argentina showed the best seed germination rate under 220 mM NaCl [31]. A recent study showed that one salt-tolerant and productive line (C2, renamed as Zhongyan 1) was screened from seven clonal tall wheatgrass lines in coastal saline–alkaline soils with salinity of 0.3% and 0.5% (*w*/*w*) [44]. As the results from field and germination tests were somewhat inconsistent, evaluations based on both germination and field performance may improve selection efficiency [4]. In this study, a total of 28 tall wheatgrass lines were evaluated by integrating seed germination under both 250 mM NaCl and 100 mM Na_2_CO_3_ stress, seedling emergence under 250 mM NaCl, and seedling growth under 300 mM NaCl. Five salt-tolerant lines with higher biomass, better seed germination, and higher seedling emergence rate under salt stress were selected, which could be utilized in saline–alkaline land. Additionally, two *Tritipyrum* lines (Lines 11 and 122) exhibited high relative biomass under salt stress, which also have potential as forage crops in the “Coastal Grass Belt” targeted region. The integrated method for salt tolerance evaluation improves selection efficiency by eliminating most salt-sensitive lines. In practice, the herbage yield potential of salt-tolerant lines should be evaluated though multi-location and multi-year trials.

## 4. Materials and Methods

### 4.1. Plant Materials

Seeds from the tall wheatgrass line Zhongyan 1 and 28 TWG lines with diverse agronomical traits were used in this work. In addition, five BC_3_F_3_ *Tritipyrum* lines, 11, 21, 94, 105, and 122, derived from the distant hybridization between wheat cv. Xinong 6028 and tall wheatgrass line Zhongyan 1 were included to investigate the transfer of salt tolerance of tall wheatgrass to wheat.

### 4.2. Evaluation of Seed Germination of Tall Wheatgrass Under Salt Stress

Different concentrations of NaCl (100, 150, 200, 250, and 300 mM), Na_2_SO_4_ (50, 100, 150, 200, and 250 mM), NaHCO_3_ (50, 100, 150, 200, and 250 mM), and Na_2_CO_3_ (25, 50, 75, 100, and 125 mM) were used to evaluate seed germination in tall wheatgrass under salt stress. Deionized water was used as the non-stress control. Seed germination tests were conducted in sterile plastic cultural dishes (diameter = 90 mm). Two layers of sterile filter paper (diameter = 90 mm) were soaked in distilled water or salt solution before sowing the seeds. In total, 50 seeds were tested in each culture dish and three independent replicates per treatment. The seeded culture dishes were kept in a growth chamber at 23 °C (day)/20 °C (night), with a 12 h photoperiod, relative humidity of 65–70%, and a photosynthetic photon flux density (PPFD) of 160 µmol m^−2^ s^−1^. Germinated seeds were counted daily from 3 to 15 DAT. Throughout the seed germination test period, deionized water was added as needed to compensate for evaporation and maintain the water volume.

The seed germination traits were computed as follows:

Germination rate = (number of germinated seeds/50) × 100%;
Germination potential = (number of germinated seeds at 4 DAT/50) × 100%;
Germination index = (sum of germinated seeds at 15 DAT)/15;
Vigor index = germination index × mean shoot height;
Relative salt damage rate = [(germination rate of control − germination rate of treatment)/germination rate of control] × 100%.

### 4.3. Evaluation of Seedling Emergence of Tall Wheatgrass Under Salt Stress

A total of 30 seeds were sown in plastic pots (80 × 80 × 80 mm) filled with sterile vermiculite and irrigated with 1 L of 0, 100, 150, 200, or 250 mM NaCl solution or saline water for 15 d. According to Li et al. (2023) [24], the saline water with EC_w_ = 8.2 dS m^−1^ and pH = 9.6 was collected from a nearby drainage ditch at the Agricultural Experiment Station for Saline–Alkaline Land in the Yellow River Delta Region (118°84′03″ E, 37°68′74″ N), Institute of Genetics and Developmental Biology, Chinese Academy of Sciences. It was diluted with deionized water to EC_w_ = 0.6, 4.9, 5.4, 6.0, 6.6, and 7.5 dS m^−1^ before use. SER was recorded daily from 4 to 7 DAT and every two days from 7 to 15 DAT. Additionally, shoot height, SFW, and SDW were determined at 15 DAT. At the end of the experiment, the salinity in the vermiculite was measured using an activity meter (PNT3000, STEP systems, Nürnberg, Germany).

### 4.4. Evaluation of Seedling Growth of Tall Wheatgrass Under Salt Stress

Seeds were germinated on a nylon net in deionized water at room temperature. Until the shoot reached 8–10 cm, 7 days after sowing, uniform seedlings were selected and transferred into a nutrition medium, which was refreshed every three days. The solution medium consisted of 0.2 mM KH_2_PO_3_, 0.5 mM MgSO_4_, 1.5 mM KCl, 1.5 mM CaCl_2_, 0.1 mM FeEDTA, 2 mM Ca(NO_3_)_2_, 1 × 10^−3^ mM H_3_BO_3_, 1 × 10^−4^ mM (NH_4_)_6_Mo_7_O_24_, 5 × 10^−4^ mM CuSO_4_, 1 × 10^−3^ mM ZnSO_4_, and 1 × 10^−3^ mM MnSO_4_. Three days later, NaCl was added to the nutrition medium to achieve final concentrations of 150, 200, 250, 300, and 400 mM NaCl, with zero NaCl addition serving as the non-stress control. After culturing in a growth chamber for 15 d, the NaCl stress was removed, and the seedlings were cultured in the nutrition medium for an additional 15 d to evaluate growth recovery. During plant culture period, the PPFD was set at 160 µmol m^−2^ s^−1^, the photoperiod was 12 h, the temperature was maintained at 24–26 °C (day) and 20–21 °C (night). The relative humidity was kept between 60% and 65%.

In addition, a daily stepwise increase of 50 mM to a final concentration of 300 mM NaCl, as well as sudden stress of 300 mM NaCl for 15 and 20 d, were performed to compare the effects of sudden and stepwise-increased salt stress on seedling growth in tall wheatgrass. Furthermore, to investigate the effects of seedling age on salt tolerance, 7 d, 14 d, 21 d, and 27 d seedlings were subjected to 0 and 400 mM NaCl for 15 d. Finally, to study the effects of growth light intensity on salt tolerance, 7 d seedlings were subjected to 250 and 500 mM NaCl stress for 15 d under growth light intensities of 100 and 300 µmol m^−2^ s^−1^. Other growth conditions remained unchanged, as described above. After the treatments, TFW, SFW, RFW, TDW, SDW, RDW, shoot height, root length, tiller number, the number of senescent leaves, and the number of green leaves were measured and analyzed.

### 4.5. Total RNA Extraction and First-Strand cDNA Synthesis

Leaf and root samples from hydroponic culture experiment were used to extract total RNA and synthesize the first-strand cDNA for assay of gene expression. According to the manufacturer’s instructions, total RNA was extracted with the TRIzol Reagent (Thermo Fisher Scientific, Waltham, MA, USA). RNA concentration was quantified by using a spectrophotometer Nanodrop 2000 (Thermo Fisher Scientific, Waltham, MA, USA). Genomic DNA was removed with a TransScript One-Step gDNA Removal kit, and 2 μg of total RNA was used to synthesize the first-strand cDNA with the cDNA Synthesis SuperMix kit (TransGen Biotech Co., Ltd., Beijing, China). The 20 μL of cDNA solution was diluted to 60 μL with H_2_O before being used for gene expression analysis.

### 4.6. qPCR Assay for Relative Gene Expression

Gene-specific primers for 23 salt-response genes and an internal reference actin gene, *ACT4*, were designed based on the full-length cDNA sequences of Zhongyan 1, which comprised over 50,000 unique isoforms by using Primer3web (version 4.1.0). The gene annotations and specific qPCR primer sequences are shown in Table S2. The full-length cDNA sequences used for primer design are listed in Table S3. Quantitative real-time polymerase chain reaction (qPCR) was conducted by using a StepOnePlus^TM^ Real-Time PCR System (Thermo Fisher Scientific, Waltham, MA, USA) to study the relative gene expression. The qPCR reaction solution included 1.5 μL of cDNA template, 5 μL of 2× PowerUp SYBR Green Master Mix (Thermo Fisher Scientific, Waltham, MA, USA), and 0.3 μL of 10 μM gene-specific primers, resulting in a final volume of 10 μL. For each sample, four technique and three biological replicates were performed. Relative expression levels were analyzed using the *C*_T_ relative quantification method [68]. Mean relative expression values were used for gene cluster analysis with the pheatmap package (version 1.0.12) in R (version 4.2.2) [69]. Rows (samples) were scaled, and the clustering distance was calculated using the correlation method.

### 4.7. Tall Wheatgrass Lines and Tritipyrum Lines Evaluation

Seeds from a total of 28 tall wheatgrass lines were germinated as mentioned above in 250 mM NaCl and 100 mM Na_2_CO_3_ for 9 d, respectively. In addition, seeds were sown in vermiculite fully irrigated with nutrient solution supplemented with 250 mM NaCl for 10 d. Three independent experiments were carried out. Deionized water or zero NaCl addition was taken as control, and the relative seed germination and SER were computed. Finally, the 28 tall wheatgrass lines were hydroponically cultured and exposed to 300 mM NaCl for 15 d. Considering energy input and evaluation efficiency, 160 µmol m^−2^ s^−1^ growth light intensity, 7 d seedlings of tall wheatgrass, and sudden salt stress of different concentrations of NaCl were used for salt tolerance evaluation in tall wheatgrass breeding programs. For each line, a half of sixteen plants were cultured in two plastic boxes as replicates. Finally, TFW, SFW, RFW, TDW, SDW, and RDW were determined. Also, five *Tritipyrum* lines, as along with the female tall wheatgrass parent Zhongyan 1 and the male wheat parent Xinong 6028, were hydroponically cultured in 250 mM NaCl for 15 d and evaluated as described above.

### 4.8. Statistical Analysis

One-way analysis of variance (ANOVA), multiple comparisons, and the least significant difference (LSD) calculations were conducted by using the SPSS (version 19.0, IBM, Armonk, NY, USA) software package. Data were shown as mean ± standard error (SE). The figures were depicted by using Graphpad Prism (version 9.0, GraphPad Software, San Diego, CA, USA).

## 5. Conclusions

In this work, seed germination, seedling emergence, and seedling growth were evaluated to assess salt tolerance in tall wheatgrass. Seed germination tests showed that exposure to 250 mM NaCl and 100 mM Na_2_CO_3_ for 9 d resulted in relative germination rates of 31.5% and 32.6%, respectively, compared to the non-stress control. Furthermore, when subjected to 250 mM NaCl and saline water with EC_w_ = 6.6 dS m^−1^ for 9 d, the relative seedling emergence rates were 52% and 59.8%, respectively, relative to the non-stress control. Hydroponic experiments revealed that 7 d seedlings exposed to sudden salt stress of 300 mM NaCl for 15 d under a growth light intensity of 160 µmol m^−2^ s^−1^ could effectively evaluate salt tolerance in tall wheatgrass at the seedling stage. This stress treatment resulted in relative TDW, SDW, and RDW of 38.2%, 35.7%, and 50%, respectively, compared to the non-stress control. An integrated evaluation of seed germination, seedling emergence, and seedling growth in twenty-eight tall wheatgrass lines under 300 mM NaCl stress identified five lines exhibiting salt tolerance. Additionally, based on seedling growth performance under 250 mM NaCl stress, two *Tritipyrum* lines, 11 and 121, seemed to inherit salt tolerance from Zhongyan 1. The five tall wheatgrass lines and two *Tritipyrum* lines have the potential to be used in the coastal saline–alkaline land around the Bohai Sea. The forage productivity and adaptability of these salt-tolerant lines should be further assessed under field conditions.

## Figures and Tables

**Table 1:** The mean values of seed germination, seedling emergence, and seedling growth traits in 28 tall wheatgrass lines responding to salt stress.

Lines	Seed Germination Test										RER (%)	Seedling Growth Test					
	RGR (%)		RDR (%)		RGI (%)		RSH (%)		RVI (%)			SFW (g)	RFW (g)	TFW (g)	SDW (g)	RDW (g)	TDW (g)
	NaCl	Na_2_CO_3_	NaCl	Na_2_CO_3_	NaCl	Na_2_CO_3_	NaCl	Na_2_CO_3_	NaCl	Na_2_CO_3_							
TWG8	43.5	28.2	56.5	71.8	15.0	11.9	23.5	18.9	2.2	0.9	118.5	0.168	0.100	0.268	0.036	0.011	0.046
TWG216	50.4	30.7	49.6	69.3	18.6	12.7	29.0	20.0	4.4	2.7	96.6	0.104	0.056	0.160	0.024	0.008	0.031
TWG223	45.6	44.1	54.4	55.9	19.7	18.4	24.7	25.3	4.2	3.5	85.7	0.123	0.085	0.208	0.026	0.010	0.036
TWG86	45.2	15.9	54.8	84.1	17.4	6.8	31.8	15.6	5.9	0.9	96.7	0.152	0.082	0.234	0.032	0.009	0.041
TWG22	38.5	13.9	61.5	86.1	15.2	5.6	21.9	13.1	3.2	0.8	104.9	0.164	0.092	0.255	0.035	0.010	0.045
Group 1	44.7 a	26.6 a	55.3 b	73.4 b	17.2 a	11.1 a	26.2 a	18.6 a	4.0 a	1.8 a	100.5 a	0.142 a	0.083 a	0.225 a	0.030 a	0.009 a	0.039 a
TWG31	49.6	10.9	50.4	89.1	19.6	4.5	30.2	9.3	5.9	0.5	57.0	0.116	0.055	0.171	0.023	0.008	0.031
TWG90	48.0	11.2	52.0	88.8	17.9	4.3	26.2	10.2	5.1	0.3	54.2	0.140	0.086	0.226	0.028	0.009	0.037
TWG182	48.6	11.4	51.4	88.6	20.1	4.8	28.3	8.6	5.3	0.8	52.9	0.101	0.063	0.163	0.023	0.008	0.030
TWG48	39.5	17.1	60.5	82.9	19.4	9.3	21.5	8.0	4.9	1.0	60.4	0.085	0.042	0.126	0.018	0.005	0.023
TWG88	38.6	8.7	61.4	91.3	16.1	4.0	27.4	6.4	3.8	0.2	54.0	0.160	0.093	0.252	0.035	0.011	0.046
TWG30	48.1	26.7	51.9	73.3	21.3	10.4	37.0	17.5	6.0	1.5	64.1	0.139	0.066	0.204	0.032	0.009	0.040
TWG209	42.1	36.4	57.9	63.6	16.6	14.7	31.9	30.1	4.8	4.1	43.0	0.126	0.076	0.202	0.028	0.011	0.039
TWG214	42.7	32.1	57.3	67.9	16.8	15.6	27.9	24.7	4.9	2.5	39.7	0.116	0.064	0.179	0.024	0.007	0.031
Group 2	44.7 a	19.3 ab	55.3 bb	80.7 ab	18.5 a	8.4 a	28.8 a	14.3 ab	5.1 a	1. 4ab	53.2 b	0.123 ab	0.068 a	0.190 ab	0.026 ab	0.009 a	0.035 ab
TWG9	34.3	5.7	65.7	94.3	14.3	2.7	15.3	2.1	4.2	0.1	20.3	0.142	0.083	0.224	0.029	0.009	0.038
TWG15	29.9	16.8	70.1	83.2	10.4	6.3	18.0	15.6	2.1	0.9	44.6	0.089	0.055	0.144	0.021	0.007	0.028
TWG65	9.9	3.3	90.1	96.7	4.2	1.2	9.6	2.2	0.4	0.0	50.3	0.122	0.071	0.192	0.025	0.007	0.032
TWG105	15.2	11.6	84.8	88.4	5.4	4.9	11.0	13.3	0.3	0.7	16.5	0.138	0.079	0.218	0.030	0.011	0.040
TWG106	13.8	2.3	86.2	97.7	5.8	1.0	10.0	1.1	0.6	0.0	22.8	0.110	0.067	0.177	0.023	0.007	0.029
TWG164	18.4	5.7	81.6	94.3	7.4	2.3	18.6	5.4	1.4	0.2	22.2	0.108	0.063	0.171	0.023	0.008	0.030
TWG180	15.8	18.7	84.2	81.3	6.3	8.0	10.6	14.1	0.8	1.0	28.1	0.127	0.086	0.212	0.027	0.009	0.035
TWG185	13.9	10.2	86.1	89.8	5.1	4.2	11.0	10.5	0.6	0.4	30.5	0.115	0.048	0.163	0.023	0.006	0.028
TWG186	26.3	1.7	73.7	98.3	9.5	0.6	17.3	2.0	1.5	0.0	12.6	0.116	0.062	0.177	0.025	0.008	0.032
TWG213	27.3	16.4	72.7	83.6	10.2	6.2	19.8	12.1	2.1	0.7	9.3	0.097	0.054	0.151	0.021	0.008	0.029
Group 3	20.5 b	9.2 b	79.5 a	90.8 a	7.9 b	3.7 b	14.1 b	7.8 b	1.4 b	0.4 b	25.7 c	0.116 b	0.067 a	0.183 b	0.025 b	0.008 a	0.032 b
TWG52	68.5	58.7	31.5	41.3	28.1	28.0	42.4	31.5	11.8	7.6	92.6	0.137	0.083	0.219	0.028	0.010	0.038
TWG70	67.7	40.0	32.3	60.0	30.9	18.5	32.0	22.1	10.9	3.9	103.7	0.093	0.055	0.148	0.022	0.009	0.031
Group 4	68.1	49.35	31.9	50.65	29.5	23.25	37.2	26.8	11.35	5.75	98.2	0.115	0.069	0.1835	0.025	0.010	0.035
TWG40	50.6	8.0	49.4	92.0	20.7	3.1	22.2	1.5	5.7	0.0	82.8	0.113	0.067	0.180	0.028	0.010	0.037
TWG157	64.9	2.1	35.1	97.9	29.2	1.0	26.2	1.1	8.4	0.0	108.7	0.122	0.079	0.201	0.027	0.014	0.041
Group 5	57.8	5.1	42.3	95.0	25.0	2.1	24.2	1.3	7.05	0.0	95.8	0.118	0.073	0.191	0.028	0.012	0.039
TWG206(Group 6)	27.7	48.2	72.3	51.8	9.5	20.3	15.4	24.3	1.2	4.6	24.1	0.123	0.052	0.174	0.026	0.006	0.032

Notes: The seed germination test was carried out in 250 mM NaCl and 100 mM Na_2_CO_3_ for 9 d, while the seedling emergence test was carried out in 250 mM NaCl for 10 d. In addition, the seedling growth test was conducted with 300 mM NaCl for 15 d. RGR, relative seed germination rate; RDR, relative salt damage rate; RGI, relative seed germination index; RSH, relative shoot height; RVI, relative vigor index; RER, relative seedling emergence rate; SL, the number of senescent leaves; GL, the number of green leaves; SFW, shoot fresh weight; RFW, root fresh weight; TFW, total fresh weight; SDW, shoot dry weight; RDW, root dry weight; TDW, total dry weight; R/S, the ratio of root to shoot dry weight. Data are represented as mean (n = 3 for germination and seedling emergence test; n = 8 for seedling growth test). One-way ANOVA analysis and LSD multiple comparison were performed for the first three groups. The different letters indicate the significant difference at *p* < 0.05 among the three groups.

## Data Availability

Data are contained within the article or Supplementary Material. Raw data are available by contacting the corresponding author.

## Supplementary Materials

The following supporting information can be downloaded at https://www.mdpi.com/article/10.3390/plants14070983/s1: Figure S1: Dynamic response of survival rate of Zhongyan 1 to different concentration of NaCl; Figure S2: Comparison of salt tolerance in tall wheatgrass line Zhongyan 1 at different seedling age; Figure S3: Hierarchical cluster analysis of 28 tall wheatgrass lines according to salt tolerance combining seed germination rate, seedling emergence rate, and seedling growth traits; Figure S4: Comparison of the effects of non-stress control and salt stress resulting from stepwise increase and sudden increase to 300 mM NaCl on seedling growth of tall wheatgrass line Zhongyan 1; Table S1: The germination traits of tall wheatgrass line Zhongyan 1 subjected to different concentrations of salts; Table S2: The sequences of gene specific primers used for qPCR in this work; Table S3: cDNA sequences for primer designing in this work.

## Abbreviations

The following abbreviations are used in this manuscript:

DAT	Days after treatment
RDW	Root dry weight
RFW	Root fresh weight
SDW	Shoot dry weight
SER	Seedling emergence rate
SFW	Shoot fresh weight
TDW	Total dry matter weight
TFW	Total fresh matter weight
