# Supplementary material for: An Integrated Method for Evaluation of Salt Tolerance in a Tall Wheatgrass Breeding Program
Source: Plants (Basel). 2025 Mar 22;14(7):983. doi: 10.3390/plants14070983 (PMC11990777; doi:10.3390/plants14070983)
Supplement: Supplementary file 1 [file plants-14-00983-s001.zip › plants-3511244-supplementary.pdf]

**Supplementary Materials**

**Table S1.** The germination traits of tall wheatgrass line Zhongyan 1 subjected to different concentrations of salts.

| Salt | Concentration (mM) | Germination potential (%) | Germination index (%) | Relative salt damage rate (%) | Vigor index | Shoot height (cm) | Percent inhibition of non-stress control (%) |
|---|---|---|---|---|---|---|---|
| NaCl | 0 | 82.0 ± 5.3 a | 12.4 ± 0.6 a | 0 | 193.0 ± 4.4 a | 15.6 ± 0.4 a | - |
| | 100 | 44.0 ± 3.5 b | 9.9 ± 0.3 b | -3.8 ± 2.4 b | 126.1 ± 5.8 b | 12.8 ± 0.2 b | 17.9 |
| | 150 | 3.3 ± 1.2 c | 7.9 ± 0.3 c | -1.5 ± 6.1 b | 57.3 ± 9.4 c | 7.3 ± 1.0 c | 53.2 |
| | 200 | 0 | 5.4 ± 0.4 d | 1.6 ± 3.5 b | 28.7 ± 3.7 d | 5.3 ± 0.4 d | 66.3 |
| | 250 | 0 | 4.1 ± 0.2 e | 3.9 ± 3.5 b | 16.8 ± 3.6 e | 4.1 ± 0.8 e | 73.5 |
| | 300 | 0 | 0.9 ± 0.2 f | 73.1 ± 7.4 a | 1.2 ± 0.2 f | 1.4 ± 0.2 f | 91.0 |
| $Na_2SO_4$ | 0 | 82.0 ± 4.0 a | 12.6 ± 0.6 a | 0 | 200.8 ± 13.0 a | 16 ± 0.3 a | - |
| | 50 | 66.0 ± 2.0 b | 10.7 ± 0.1 b | 2.3 ± 2.6 c | 137.3 ± 8.9 b | 12.8 ± 0.7 b | 19.8 |
| | 100 | 11.3 ± 16.3 c | 7.7 ± 0.9 c | 6.8 ± 4.7 c | 72.7 ± 8.1 c | 9.4 ± 0.1 c | 40.9 |
| | 150 | 0 | 4.9 ± 0.5 d | 7.5 ± 3.9 c | 33.6 ± 5.7 d | 6.8 ± 0.6 d | 57.6 |
| | 200 | 0 | 2.1 ± 0.4 e | 48.1 ± 9.9 b | 8.0 ± 0.4 e | 4.0 ± 0.7 e | 75.2 |
| | 250 | 0 | 0 ± 0 | 100.0 ± 0.0 a | 0 | 0 | 100.0 |
| $NaHCO_3$ | 0 | 82.0 ± 4.0 a | 12.6 ± 0.6 a | 0 | 200.8 ± 13.0 a | 16.0 ± 0.3 a | - |
| | 50 | 76.0 ± 6.0 a | 11.7 ± 1.1 b | 0.8 ± 6.0 d | 144.1 ± 13.2 b | 12.3 ± 0.3 b | 23.0 |
| | 100 | 48.0 ± 5.3 b | 9.3 ± 1.0 c | 4.5 ± 5.7 d | 71.8 ± 10.8 c | 7.7 ± 0.4 c | 51.7 |
| | 150 | 0.7 ± 1.2 c | 5.1 ± 0.4 d | 21.8 ± 7.3 c | 34.5 ± 4.2 d | 6.7 ± 0.5 d | 58.1 |
| | 200 | 0 | 1.6 ± 0.2 e | 67.7 ± 2.6 b | 6.6 ± 1.3 e | 4.2 ± 0.4 e | 73.6 |
| | 250 | 0 | 0.1 ± 0.1 f | 97.0 ± 2.6 a | 0 ± 0.1 e | 0.3 ± 0.3 f | 98.1 |
| $Na_2CO_3$ | 0 | 82.0 ± 5.3 a | 12.4 ± 0.6 a | 0 | 193 ± 4.4 a | 15.6 ± 0.4 a | - |
| | 25 | 68.0 ± 4.0 b | 10.2 ± 0.4 b | 0.8 ± 2.3 d | 144.6 ± 15.4 b | 14.2 ± 0.9 b | 8.8 |
| | 50 | 12.0 ± 7.2 c | 8.3 ± 0.3 c | 3.9 ± 4.8 d | 83.0 ± 2.3 c | 10.0 ± 0.1 c | 35.7 |

| | | | | | | |
|---|---|---|---|---|---|---|
| 75 | 0 | 4.7 ± 0.4 d | 27.0 ± 1.3 c | 30.7 ± 3.0 d | 6.5 ± 0.1 d | 58.1 |
| 100 | 0 | 2.5 ± 0.2 e | 51.5 ± 2.3 b | 10.6 ± 1.9 e | 4.1 ± 0.4 e | 73.4 |
| 125 | 0 | 0.1 ± 0.1 f | 97.7 ± 2.3 a | 0 | 0 | 100.0 |

Notes: Data are represented as mean ± SE (n=6). Different letters indicate that the difference was significant at $p < 0.05$.

**Table S2.** The sequences of gene specific primers used for qPCR in this work.

| Genes | Forward sequence | Reverse sequence | Product (bp) | Gene annotation |
|---|---|---|---|---|
| *ACT4* | CTAGTGGACGCACAACAGGT | AAGGATGGCATGTGGAAGGG | 98 | Actin |
| *APX4* | AGCCCAAACACACCTCAGAC | CAACGCGGATGACCCTAGAA | 80 | Probable L-ascorbate peroxidase 4 |
| *BASS2* | TGAGTGTGGGATGCAGAGTT | CCATGCACACAACACTGACA | 104 | Probable sodium/metabolite cotransporter BASS2 |
| *BASS3* | CGTATTGGGGAAGTGGCATG | CCCAAATGGCAGCAGAGTTT | 149 | Probable sodium/metabolite cotransporter BASS3 |
| *BASS4* | GGTCATCCCATGTGTTGCTG | GTCAAGCCTCACTCGGATAGA | 157 | Probable sodium/metabolite cotransporter BASS4 |
| *BASS5* | ACAGGGATGCAAAGTAGCCT | TCAGGGACATCAGCACAACT | 106 | Probable sodium/metabolite cotransporter BASS5 |
| *DHAR1* | GCCTTGATGTGCTGTGGTTG | CCGCTAGCACCAAAACACAC | 116 | Probable glutathione S-transferase DHAR1 |
| *ESI3* | AGCCTCGAGTGAAGTAAGCA | GAATTCCACGCCGAGTTTGT | 114 | Salt stress-induced hydrophobic peptide ESI3 |
| *GPX1* | GAGTCGAGTGCTGGAGGATT | CCGCAACGAGTTTCTGGATG | 148 | Phospholipid hydroperoxide glutathione peroxidase 1 |
| *GPX6* | TCGCTTCAAGGCCGAGTATC | CCACTTGATGTTGTCCCCGA | 121 | Phospholipid hydroperoxide glutathione peroxidase 6 |
| *GR* | AATACAGAGCTGCAACGCCT | ACCTTTCCACGGCCTTCAAT | 86 | Glutathione reductase |
| *LOX23* | GACCAGCGAAACAACAACCC | CTTATCCTGGGGCCACGATC | 147 | Lipoxygenase 2.3 |
| *NCED1* | TCAGACACACCAACACGTCC | CCTACCCATGCACCACTGTT | 88 | 9-cis-epoxycarotenoid dioxygenase 1 |

| | | | | |
|---|---|---|---|---|
| *NCL1* | GACCATGAACAACACGCTGT | ATGACACAGACGAGCAGGAT | 111 | Sodium/calcium exchanger-like protein NCL1 |
| *NCL2* | ATCTGGTACGTGTTTCCGGT | CAGCAACACACGCGATATCA | 116 | Sodium/calcium exchanger-like protein NCL2 |
| *NHD1* | TGTTCCACTTGTTGCTGCAA | TGCAGACCCGATGATAAGCA | 127 | Sodium/proton antiporter NHD1 |
| *NHX1* | TTATTGTGCTGCTGTGACCA | CGACCTCCCTTTTCCATGTG | 97 | Sodium/hydrogen exchanger NHX1 |
| *NHX2* | TGGTGCGATGGGGCTAATAT | GCCGAGAATATTGCCCCAAG | 84 | Sodium/hydrogen exchanger NHX2 |
| *NHX6* | CCGGCAAATATCCAGGCAAT | AAATTGTCTGTCCATGGCCG | 122 | Sodium/hydrogen exchanger NHX6 |
| *NHX7.1* | GGCTTGGGAAAATTGTCAGC | TCACCAAAAGCCTCCAATGC | 89 | SOS1 |
| *NHX7.2* | GTTTTGGCCGTCATGACCTT | GCAAACTTTCCTGGCTGTCA | 82 | SOS1 |
| *NHX8* | AGGGACGGATAAATGCAGCT | GGACACTGGACTGCAAACTG | 96 | Sodium/hydrogen exchanger NHX8 |
| *POD* | TTGCTCTGCCATTGTCCACA | GTCGATTGCCAACTGAACGG | 121 | Peroxidase 51 |
| *PSA1* | ATCGCCTCAGGTTGACAAGA | CCTCAGGCTGTTGAACGATG | 129 | Protein short root in salt medium PSA1 |

Table S3 cDNA sequences for primer designing in this work.

| Genes | Isoform ID | Sequence (5′→3′) |
|---|---|---|
| *ACT4* | C2_transcript_<br>108143 | GGAGAGGATGACACAAATCATGTTTGAGACCTTCAATGTTCCAGCCATGTATGTGGCCATTCAGGCGGTCCTTTCCCTA<br>TATGCTAGTGGACGCACAACAGGTATCGTGATGGATTCTGGTGATGGTGTCAGCCATACTGTGCCAATCTATGAAGGAT<br>ATGCCCTTCCACATGCCATCCTTCGTCTTGACCTCGCTGGGCGCGACCTAACTGACTGTTTGATGAAGATTCTTACGGA<br>GAGAGGTTACTCCTTCACCACCACTGCCGAACGGGAAATTGTAAGGGACATCAAGGAGAAGCTCGCATATGTGGCTC<br>TTGACTATGAACAAGAGCTGGAAAATGCCAAGAGCAGCTCCTCTGTGGAGAAGAGCTATGAGCTGCCTGGTGGGCA<br>GGTGATCACCATTGGGGCAGAGAGGTTCCGTTGCCCTGAGGTCCTTTTCCAGCCATCTTTCATTGGTATGGAAGCTGCT<br>GGAATCCATGAGACCACCTACAACTCTATCATGAAGTGTGATGTGGATATCAGGAAGGATCTGTATGGTAACATTGTG<br>CTCAGTGGTGGCTCAACTATGTTTCCGGGTATTGCTGACCGTATGAGCAAGGAGATCACTGCCCTTGCACCAAGCAGC<br>ATGAAGATCAAGGTGAGGGCACCGCCTGAGAGGAAGTACAGTATCTGGATTGGAGGGTCGATTCTTGCCTCCCTTAGT<br>ACCTTCCAACAGATGTGGATCTCGAAGGGCGAGTATGATGAGTCAGGTCCAGCGATTGTCCACCGGAAGTGCTTCTA<br>AGTTGCGAGTTGTCTGGGTCTTCTGAATCTATTCTGGCTGTTCTTTTCTCAAGTCCTTGCCATGTAAGCTACTATGTTTGA<br>GATTGCTGGGATTCTGTTGACTGTATTGCCGGATTAATTTGTTGGGTCAAAGTACCTTGTAGACTCGTAATATGTTCAGT<br>GGGCTGATATATTTGTCCGGTGCTGAATCGTATGCCGCATGTTCCGGGTTTTCTTTTTCATGCCAAAAAGTTTGGTGCTA<br>GCGTGGGGGGAGTGTCAAACTATCTACCAGAGTCTTTGTAAGAGTTTTCCATGCGAGTCCTATAATCATTCCCTTATGT<br>ACATTCACTTCTGCGATATGTAATCTTTTGGGCTGCCAATTTGATTC |
| *APX4* | C2_transcript_<br>138892 | GACCCTACATCAACGGTGCGAACATCTCCGCCGTTGATCTCAGCCTGGCTCCGAACCTCTAGCATCTCCAGGTCGCCC<br>TGGAGCACTTCAAAGGCTGGAAGGTCCCTGAAACCCTGACCAGCGTCCATGCCTACACCGAGGCTCTCTTCAGCCGT<br>GAGTCGTTCGTCAAGACCAAGGCGACCAAGGAGAACCTGATCGCCGGGTGGGCGCCGAAAGTGAATCCGTAAGCCC<br>TCCCGCCGCCCGGAGTCCCGACCCGCCCCCGTTGTGTGAGTAGTGATGGGCAGTAGTAGACCTCATCTTTCTAGGTTT<br>GAATAATTCGAGCCTTGATGTGCTGTGGTTGTCGCAAGCGTGCACTTGCTGCGTGGACTGTCCCCTGCCTGGCTTGTGT<br>GTGTGTGTGTGTGTGTGTGTGTGTGTGTGTGTGTTTTGGTGCTAGCGGGGGTGCAAGTATTGTGGTGTGAACCTCTTTCG<br>TATGAATGAATAATGGAGCTGCGTAAAATTCAGATTTGCCAT |
| *BASS2* | C2_transcript_<br>127701 | GCTTGGCCATCAGTACTTTTCAGGTTGTTTTAGTACCGACTATCGTTGGAGTGTTGGCACATGTGTATTTTCCCAAGTTT<br>ACCGAGCGGATTATCTCCATAACACCACTGATAGGGGTCATCCTCACCACCTTGCTTTGTGCTTGCCCTATTGGGCAAG |

| | | |
|---|---|---|
| | | TCGCAGAAGTGTTGAAAACTCAAGGTGCTCAACTTATTCCCCCTGTTGCTCTGCTGCATGCTGTTGCATTCGCTCTTGGCTATTGGATCTCGAAATGGTCTTCTTTCGGGGAATCAACTTCTAGAACCATCTCAATTGAGTGTGGGATGCAGAGTTCTGCGCTTGGATTGTTACTTGCGCAAAAACACTTCACAAACCCTCTTGTTGCTGTTCCTTCTGCTGTCAGTGTTGTGTGCATGGCGCTCGGAGGCAGTGCTCTCGCAGTTTTCTGGAGAAACAGAGGCCTTCCAGCAGATGACAAGGACGTTTTCAAGGAATGAATGAAGCAAAAACAATCCTAGGCTCCAGCTTCTAGTCGTTACCCTGTTTTAGTTGAGCGTGGTTACGTCAGAGAATTTTTTATATTCCTATATTTTAGGTATGTAAAACACAAAACATGATAAGGACGTTTGTTTATTCTAGATAATTGATTATTGGT |
| *BASS3* | C2_transcript_137214 | TCCCCGCAGCATGTTCTGTTGTTGTCATGGCAATATTTGGTTTGACTCTTGCATCGTATTGGGGAAGTGGCATGCAAATAAGAGACGTTCCTTCGAGATTCTTCCCACAAGCTTCCACTGATGCGAGATCGTGAGAGGCTGGATTATAGCATTGAAAGTTTTTTCAAGGGCTCCCAGTTTGAGAAACTCTGCTGCCATTTGGGGATGCAGTTACAAGGATGATGTCCTGCGCCTCGTCCAGCAATCAGGCATGCCATAGTACCTACAAAAGGCAGAACTACAAACCGCAGATTGTGCAAGGTAAGCAGCGGCTGGGGATAACCTCGCCACAGAAACCACTTGCATGGCTGCATACGATGGAAGGATAACAAGATGCGAGCCATTTACCACTTATCATACCATGTGCAGTAGGCTCCATTAGTGGTGCTCCCTTGGCCATTTTTCTTGTAGATACTAGATAGTAGACACGTTGACGGTTCCTATGTACACCACTAAGCATATGCATGTGCAATGCCAATATCATCGACACAGTTCTTCCC |
| *BASS4* | C2_transcript_21089 | TTGTCCGTTAAACCAAAAGCTTTCGCTATTATTGTGACTATTGGAGTGCTTCTACATTTTCTCTGCTGGCATTCAATGCTACAACACTGCAGGCCTTGTCGTGTCTTAAACAGAGGGAAGAATCGGTCTTTGCGAGGAAAGAGTATGCACGAGCTGTCATTTTGGTGGCTAGTCAGAAAACATTGCCAGTGCTGGTTGCTGTGGTTGAGCAGCTTGGAGGTGCTCTTGGGGAGTCCGGACTTCTGGTCATCCCATGTGTTGCTGCGCACATTAGTCAGATCATTATCGATTCTATCCTAGTAAATTGGTGGCGCCAAAGAGATCAACTATCTACTAGTGCAAAATAGCTTCAGAATTTCAATTTGATCTAAGTGCAAAGATCTATCCGAGTGAGGCTTGACAAATCCATGGTGAAATAGGTTTATTGGACTTGGTAGACCGCTTGGTGCACGACACGATCAGGATCCTTGTGATCCTAGAACCATTTAGTTCGAAGTCTCCATCAATGTGGAGTAGGATAACTCCAACTATCCGAACCATGAACATCGTCGCGCCCCTTATGTTAGCAATTTCTAAACTCAAACTCTTGGAAGTTATTTTTTTTGCTGCTACTGTT |
| *BASS5* | C2_transcript_69718 | GACGTCTGAACTCTGAAACCCACGCACGCAGACCTGAACCCTCTCCGCCTCCTCGCTCTTGCCGTGGTCAAATGGCCCCAGCCGCCGCCGTCGTGAGGCGGGCCCACTTCGCCAGCTCCTGCCCGTCGGCCGGCCGGCGGCTCCGCGGGAGGGCGGATCGCGTCTCGCTGGCTCCCCCCGCGGCCTCGCCGCCGCGCGTCTCATGGGAGCAGGGATCTGGGGTGCCCATCAGGGGACGAGTCTGGGCGAGGGCCGGTGGTTCGTTCGAGCAACATCGCATTCGGGTGGACGCCGCGTCAGCTTCTCC |

| | | |
|---|---|---|
| | | TCCCCAGGGGGTGGAGGAGGGCAGAGTTGATTTTCTCAAGATTCTGAAGAGTGCCAACTCCGTTATCCCCCATGTAGT<br>GCTGGGGAGCACCATTCTTGCGCTGCTGTACCCACCTTCGTTTACATGGTTCACAACCAGATACTACGCACCAGCATT<br>GGGGTTCTTGATGTTTGCTGTAGGTGTCAATTCAAGCGTTAAGGACTTCATTGAAGCAATAAAAAGGCCAGATGCTAT<br>CGCTGCGGGCTATATTGGACAGTTTATCGCCAAACCTTTCTTTGGATTCCTCTTTGGCACACTTGCAGTTAATACTTTCA<br>ATCTTCCTACTGCTGTAGGTGCTGGGATCATGTTGGTTTCATGTGTGAGCGGAGCACAATTGTCAAACTATGCAACGTT<br>CCTGACAGATCCACATATGGCTCCCCTCAGCATTGTTATGACATCACTGTCGACAGCTTGTGCGGTTTTTGTTACCCCA<br>ACTTTATCTTACTTGCTTATCGGCAAGAAATTACCTGTTGACGTCGTAGGAATGATGTCCAGCATTGTCCAAATAGTGGT<br>TGCTCCAATTGCAGCTGGATTGCTTCTGAATAGATTTCTCCCAAGACTCTGTTCGGCTATTCAGCCATTTCTCCCTCCAC<br>TATCGGTGTTTGTGACTGCTTTATGTGTTGGCTCACCATTGGCTATAAATATCAAGGCTGTTTTGTCACCATATGGACTAT<br>CCATTGTGTTTCTGCTTTTTGCATTCCATACAACATCTTTTGTAGCTGGGTATCATCTTGCTGGTACTTGGTTCCGCAAGT<br>CAGCTGATGTGAAGGCCCTGCAAAGAACGATATCTTTTGAGACAGGGATGCAAAGTAGCCTTCTTGCTCTTGCTTTAG<br>CAAACAGGTTCTTCCCAGACCCTCTGGTGGGAGTGCCTCCAGCAGTATCAGTTGTGCTGATGTCCCTGATGGGGTTTG<br>GTCTTGTGATGGTATGGTCCAAGAAAACACAAGTGTAGCCCCCGCAAAACCACTGGGCTGGATCGATGGCCGATATA<br>CTTGTCAGCGAATTTTTCGATGGCGTTGACCAAATCGCCAAGAACAACCCGATCCTTGTTGAAAGATGACGATTGCAG<br>ATCATAGATGGCCAGGGTGCCAAGTGAGAGACCAGGATCACCGTGGCACGTCTGGATCCTTGTCACTCTCTTTTCCTT<br>TGATCCTTGGTGGAAAGATGTCTGTGTAACGTTTTGGGGATTCATAGGGATAACCTCTCCCTAGTTGATTCTGACAGAA<br>GATAGAAAGGCAGAAGAAACCTACGTGCTGGTGGATTTATCATCTGATGATATCCGTTTGCATTTATTGTGACAAAAAT<br>AATAATATCCACTCATGCTGGTTATGCAGCTGTTTTTATTTTCTTAATCGAGCTGCAAACTTGGATGATGATGATGAC |
| *DHAR1* | C2_transcript_<br>138892 | GACCCTACATCAACGGTGCGAACATCTCCGCCGTTGATCTCAGCCTGGCTCCGAACCTCTAGCATCTCCAGGTCGCCC<br>TGGAGCACTTCAAAGGCTGGAAGGTCCCTGAAACCCTGACCAGCGTCCATGCCTACACCGAGGCTCTCTTCAGCCGT<br>GAGTCGTTCGTCAAGACCAAGGCGACCAAGGAGAACCTGATCGCCGGGTGGGCGCCGAAAGTGAATCCGTAAGCCC<br>TCCCGCCGCCCGGAGTCCCGACCCGCCCCCGTTGTGTGAGTAGTGATGGGCAGTAGTAGACCTCATCTTTCTAGGTTT<br>GAATAATTCGAGCCTTGATGTGCTGTGGTTGTCGCAAGCGTGCACTTGCTGCGTGGACTGTCCCCTGCCTGGCTTGTGT<br>GTGTGTGTGTGTGTGTGTGTGTGTGTGTGTGTGTGTTTTGGTGCTAGCGGGGGTGCAAGTATTGTGGTGTGAACCTCTTTCG<br>TATGAATGAATAATGGAGCTGCGTAAAATTCAGATTTGCCAT |

| | | |
|---|---|---|
| *ESI3* | C2_transcript_<br>137706 | AATCCAAATCATCCAATCCAGGCGCCTCTTTCAGAAGGGAAAGCAGAGATAGAAAGAAGCTAGACAGCCTCGAGTG<br>AAGTAAGCAAGCCATGGGCTCGGCAACAGTCCTGGAGGTGATCCTCGCCATCATCCTGCCTCCCGTCGGCGTCTTCCT<br>GCGCTACAAACTCGGCGTGGAATTCTGGATCTGTCTCTTGCTGACCATACTGGGGTACATACCGGGGATCATCTACGCG<br>GTGTACGTGCTGGTAGTTTAAGCAACAGCCTCTGCTGCAGGGTCCAGGCTTGGACGAGCGAGTCGCTGTGCAAGAGC<br>AACTGATGCTGTCTGTGCTAAGATAGCATGTCTTTGTGTATGCTTGTATGTATTGAATATTGCGGATTCCATTTTTTTGG<br>AACTCGGTATTGTTGATCCAGTGATGTCACGAGTGTACGTAGCTCGAGGGCTTTAGGCCATTCGTCGGTGTTGAGTATC<br>GTGTCTATTAGGAAATAAGAGATAGCGTAGGATTCTATTCTACCTTGCCTTGTACTCCAAGTAGACCATTGTACTTCTAT<br>ATATACGCCCACGAGGCTCGAGCAATAAACATCGATTCCATCG |
| *GPX1* | C2_transcript_<br>122163 | ACGCCACGCACCGACGCGCGGCAGCCACACCCCCCGCATCAGGGCCTCGTCCACCGCGGCCGCGGCGCTCACCTGC<br>CTCCACCCCGCCGCGCAGGCTTCGCGGCTCCCTCCGGCCGCCACCGCCTTCGCGCGCCTCCCGGCGGCCCACTCGGG<br>GTCGGGGGCCTGGGGCAGGGCGGCGGTCTCCGTCGCGGCGCCGCGGCGCCGCAGGCGGGCGCCCGGCGTCGTCTAC<br>GCCGCCGCCACCACGGAGAAGAGCATCTACGACTTCACCGTGAAGGATATTGATGGAAAAAATGTTTCTCTTAGCAA<br>GTTCAAGGGAAAAGCACGGTTGATTGTTAATGTTGCTTCTCAATGTGGGCTTACAACAGCAAATTACACTGAGCTATC<br>TCATCTTTACGAGAAGTACAAGACTCAAGGGTTTGAGATTCTGGCGTTTCCATGTAATCAATTTGGTTTTCAAGAAACT<br>GGATCAAATACACAGATAAAGCAATTTGCTTGCACAAGGTTTAAAGCTGAATTTCCTATTTTTGATAAGGTTGATGTCA<br>ATGGACCATTTACAGCCCCTATTTATAAGTTTCTCGAGTCGAGTGCTGGAGGATTTTTGGGTGGTATAGTGAAGTGGAA<br>CTTTGAAAAGTTCCTAGTGGACAAAAATGACAAAATTGTGGAGAGATACCCACCAACAACTTCACAATTCCAAATTG<br>AGAAGGACATCCAGAAACTCGTTGCGGCATAAGTGTTTTTGAGTCCAACTTCAGTTAAGCACTCCTGTATATATTGCCC<br>TTTTCCCGGAAACTAGAGTTTTACTTGTGATTATGACTCTTCTGCGATATAATGTTTTCCATTTCTGTAAAATAATTCACT<br>GATATGTATAGACATTTCCGGACTTCGG |
| *GPX6* | C2_transcript<br>_136468 | CAAGGATGCAAGTGGAAAAGATGTCGACCTGAGCACCTACAAGGGGAAGGTTCTCCTCATCGTCAATGTTGCA<br>TCCCAGTGTGGCTTGACGAATTCAAAGTACACGGAACTTGCTCAGTTGTACGAGAAGTACAAGGACCGGGGTT<br>TTGAGATCCTTGCTTTCCCATGCAACCAGTTTGGTGGGCAGGAGCCTGGCACTAATGAGGAAATTGTTCAGTTTG<br>CTTGCACTCGCTTCAAGGCCGAGTATCCAATTTTCGACAAGGTTGATGTCAACGGTGACAATGTTGCACCTGTCT<br>ACAAGTTTCTGAAGTCCAGCAAAGGCAGTCTCTTCGGGGACAACATCAAGTGGAACTTCTCCAAGTTCTTGGTT<br>GACAAGGAGGGGCGTGATGTGGATCGCTACGCGCCAACCACCTCCCCCCTCAGCATCGAGAAGGACATCAAG |

| | | |
|---|---|---|
| | | AAGCTGCTCGCGAGTTCTTAAACCTTGTTGCTGCATCAACTAGATGGATTGGTGCATCACCTGAAGCTTCAGTAA<br>TGTTGTAATAAAGTCTGTGCTCGGAACTCGCTATGTTATATATGTGCTCAGTTGCCTTGTGCAGTCCAATCTCGTA<br>GTAGTATATGGTATGTAATGGATGGTGGAATGCATTACTTCTGTTGCAGAGATATCCCCTCTATCTGTGTTTTTGTT<br>TT |
| *GR* | C2_transcript<br>_39579 | ACTTCCAGAAAGGAGCCGGCGGCAGCGCGGCGAGAGCTCGGACCTCGGAGGAAGAGGTAGCCATGGCGACC<br>ACCGCGGCCCTCCCCTTCTCCTGCGCCACCACCCTCCAGACTCTAACCCGGACCCTCTCCCCGCGCCGCTCCCT<br>CCTCATCCACCGCCACCGGCTCCGCTCCCTCGCCGCCTCCCCGCGGCTCCCGGACCGCGTCCGCCCCCGCCTCC<br>ACCGCCCCGTCTCGGCCTCCGCCGCGCCCAACGGATCCTCCTCCGCGGGGGAGTACGACTACGACCTCTTCACC<br>ATCGGCACCGGGAGCGGCGGCGTGCGGGCCTCGCGCTTCTCCTCCATCCTCTACGGCGCCCGCGCCGCCATCTG<br>CGAGATGCCCTTCTCCACCATCTCCGCGGACGACCTCGGCGGCCTCGGGGGGACATGTGTGCTTCGTGGGTGTG<br>TTCCGAAGAAACTGTTAGTGTATGCATCCAAGTTCTCTCATGAGTTTGAAGAGTCTCACGGCTTTGGATGGACAT<br>ATGATACTGATCCAAAACATGACTGGAGTACTCTGATAGCCAACAAAAATACAGAGCTGCAACGCCTAGTTGGC<br>ATTTACAAAAATATTTAAACAACGCAAACGTCGATCTAATTGAAGGCCGTGGAAAGGTGGTTGATCCACATAC<br>TGTTAGTGTGGATGGCAAGCTCTACACCGCTAAGAACATACTTATAGCTGTTGGTGGTCGACCATCGATGCCAGA<br>TATCCCAGGAATAGAGCATGTCATAGATTCCGATGCTGCACTAGATCTGCCTTCAAAACCTGAGAAAATTGCAAT<br>AGAGGGAGGTGGATATATTGCCTTGGAGTTTGCTGGCATTTTTAATGGCTTAAAAAGTGACGTTCATGTGTTTATT<br>CGGCAACCGAAAGTTTTAAGAGGGTTTGATGAGGAGGTCAGAGATTTCGTTGCTGAACAGATGTCTTTAAGGGG<br>TATCACATTTCATACTGAACATAGTCCTCAAGCTATAACCAAATCAAATGATGGTTTACTATCTCTGAAGACAAA<br>CAAAGAGACTATTGGTGGGTTTTCACATGTAATGTTCGCAACAGGTCGTAAACCGAATACGAAGAACCTCGGAC<br>TGGAGGAGGTTGGAGTCAAAATGGACAAGAAGGGGGCTATAGTGGTCGATGAGTATTCTCGTACCTCAGTGGAT<br>TCAATTTGGGCTGTTGGAGATGTTACTGATAGGATCAACTTGACTCCGGTTGCACTGATGGAAGGTGAAGCATTT<br>GCGAAAACTTTATTCGGTGACGAACCTACCAAACCAGAGTACAGAGCTGTACCAGCTGCTGTTTTCTCCCAACC<br>ACCCATTGGGCAAGTTGGCCTTACCGAGGAGCAGGCTATTGAAGAGTATGGAGATGTTGATGTCTACTTGTCAA<br>ACTTCAGACCTCTTAGAGCCACTCTTTCTGGATTACCTGATCGTGTACTAATGAAGCTCATTGTGTGTGCTACGAC<br>GAACAAAGTTGTAGGAGTGCATATGTGCGGTGATGATGCACCTGAGATAATCCAGGAATTGCAATTGGTGTTAA<br>AGCCGGGTTAACGAAGCAAGATTTTGATGTCACTGTTGGCGTTCACCCAACATCTGCAGAGGAATTTGTCACTA |

| | | |
|---|---|---|
| | | TGAGGAGTCCAACTAGAAAAGTTCGAAGAAAAACTGCAACTGAGAGTCTAAGGATGAGGTCGTCACTCAGAAGTAGATCATTGAGCAATGGTGCCCGGCATCATTGTATTGATCTTGATTAACCAAATCATCAACAATAGTTGCAAACCCCGTCTTTGACTAGTTGAGTTTTCTTATCAGTCATTCTCGAGGATGCCATGCCACCGGCCAAAACCTGACTGGAAATACACAGCTAAACAGTGTGCACCGTTGCCTGAGAAATCTTTCAGTGGACCTCGGAAGATGTACGATAACGATGAGTTGCTACCTCAGTTTTTTTGCCCTCCCATTTGCACACTTCTTTCCTTCCTGCTTGAGGCTTGAGCACGCTGGTCGTTGAGTTTCTTTTCCCCATCTTGCTATTCATGTGTAATAAATTGTACAATGTGGCTGTGTGGGTATCATCAACCTTGAAGCATCGATCCGGAATACTACCAGAAAACCAGTGTATAGAGTTTAAATACCTGCAGATCGATCTGCGACCAGTT |
| *LOX23* | C2_transcript_76568 | GTTCTGGAGAAGATGATGAACGGGCGCATGACGGTGGAGGAGGCGGTGGAGAAGAAGCGGCTGTACCTGCTGGACTACCACGACGTGTTCCTGCCGTACGTGCACAGGGTGCGCGACCTGCCGGACACGACGCTGTACGGGTCCCGCACCGTCTTCTTCCTGAGCGATGAGGGCACGCTGATGCCGCTGGCCATCGAGCTGACGCGGCCACAGTCGCCGACCAAGCCGCAGTGGAAGCGCGCCTTCACGCACGGCTCCGACGCCACCGAGTCGTGGCTATGGAAGCTGGCCATGGCGCACGTGCTGACCCACGACACCGGCTACCACCAGCTGGTCAGCCACTGGCTGCGCACGCACGTCTGCGTCGAGCCCTACATCATCGCCACCAACCGGCAGCTCAGCCGGATGCACCCGGTGCACCACCTGCTGCACCCGCACTTCCGCTACACCATGGAGATCAACGCGCTGGCCAGGGAGTCCCTCATCAACGCCGACGGCATCATCGAGGAGGCATTCTGGCGGGGGAGGTACTCCATCGAGCTCAGCTCCGTCGCCTACGGCGCCGCGTGGCAGTTCAACACGGAGGCGCTGCCGGAGGACCTCGTCAGCCGGGGACTCGCCGTGCGCAGGGACGACGGCGAGCTCGAGCTCGTCATCAAGGACTACCCGTACGCCGACGAGGGGCTGCTGATCTGGGGCTCCATCAAGGAGTGGGCGTCCGACTACGTGGACGTCTACTACAAGTCGGACGAGGACGTCGCCGGCGACGAGGAGCTGCGGGCGTGGTGGAGGAGGTGCGCACCAAGGGGCACGCGGACAAGAAGGACGAGCCGTGGTGGCCCGTGTGTGACTCCAAGGAGAAGCTCGTTCAGATCCTGACCACCATCATGTGGGTCACGTCCGGCCACCACGCCGCCGTCAACTTCGGGCAGTACCATTACGCCGGGTACTTCCCCAACCGCCCGACCGTGGTGCGGAGGAACATCCCGGTGGAGGAGAACCGGGACGACGAGATGAAGAAGTTCATGGCCAGGCCGGAGGAGGTGCTGCTGCAGAGTCTGCCCTCACAGATGCAGGCCATCAAGGTGATGGCGACGCTGGACATCCTCTCCTCGCACTCCCCCGACGAGGAGTACATGGGAGAGTACGCTGAGCCGGCGTGGCTGGCTGAGCCCATGGTGAAGGCGGCGTTCGAGAAGTTCAGCGGCAGGCTCAAGGAGGTGGAGGGCACCATCGACCAGCGAAACAACAACCCGGAGAACAAGAACAGGTGTGGCGCCGGCATCGTGCCGTACGAGCTGCTCA |

| | | |
|---|---|---|
| | | AACCGTTCTCAGAACCAGGGGTCACCGGGAGGGGCATCCCCAACAGCATCTCCATCTGATCGTGGCCCCAGGA<br>TAAGATATCATTGCATGTACGATTAGGAATAAGATGTACACCATGTACGCACCCAGCAGCGATTGCTCTAGGACA<br>TGACACGGCCCGGGAGAGGCATCTACTAGTGTGTTCCCGCTATTGTATGCCGTGAATTAATAATACTAATAAGAT<br>TATTTGAATTGTCAGCATGGTTAGTTTTATCGGTTGTAACATTACTCACTCTAGAAAGAGTGGGCATCTTCGTGCA<br>TTCATGGACAGATCATATTGTACCGTAGC |
| *NCED1* | C2_transcript<br>_62244 | GCGGCGTTCTGCAACGCGCTGGAGGAGGCGATTAATACGTTCGTCGACCCGCCGGTGCTGCGTCCGTCGGTGGA<br>CCCGCGGCACGTGCTGTCCGCCAACTTCGCGCCCGTGGACGAGTTGCCGCCCACGCCCTGCCCCGTGGTACGC<br>GGCGTCATCCCGCGCTGCCTCGCCGGCGGCGCCTACAACCGCAACGGGCCCAACCCGCAGCATCTCCCGCGCG<br>GGCCGCACCACCTCTTCGACGGCGACGGCATGCTGCACTCCTTGCTCCTCCCGACGACGCCCGACTCCGACCCC<br>GTCCTCTGCTCCCGCTACGTGCAGACGTACAAGTACATCGTGGAGCGCAACGCGGGCGAGCCGGTCTTGCCCA<br>ACGTCTTCTCCGGCTTCCATGGCCTGGCCGGGATGGCGCACGGCGCCGTCACGGCGGCCAGGGTGCTGACAGG<br>CCAGATGAACCCGATGGAGGGCGTGGGGCTCGCCAACACCAGCCTCGCCGTCTTCGGCGGCCGCCTCTACGCG<br>CTCGGCGAGTCGGACCTCCGTATGCCGTGCGCGTCGACCCGGCCACTGATGAGGTGACCGCGCTCGGCAGGTG<br>CGACTTCGGTGGCCGCCTCAGCATGGGCATGACTGCGCACCCCAAGAAGGACCCCATCACCGGCGAGCTCTTC<br>GCGTTCCGCTACGGACCCATGCCGCCGTTCGTCACCTATTTCCGGTTCGACCCTGCAGGGAACAAGGGCGCCGA<br>CGTGCCCATCTTCTCCGTCAAGCAACCGTCTTTCCTGCACGACTTCGCCGTGACGGAGCATTATGCGATATTCCC<br>GGAGATACAGATCGTGATGAACCCCATGGGCATGGTGGTGGGCGGCGGTTCGCCCGTCGGGGCGGACCCGAGC<br>ATGGTGCCTCGCCTCGGCGTGATCCCCAAGTACGCTGCGGACGAGTCGGAGATGCGGTGGTTCGAGGAGCCGG<br>GGTTCAACATGATGCACTCGGTGAACGCGTGGGAGGAGGCCGGCGGCGACGAGATCGTGATGGTGGCGCCCA<br>ACATCCTGTCCATCGAGCACACGATGGAGCGCATGGAGCTGATCCAGGCCTCCGTGGAGCTGGTGCGCATCAA<br>CCTCCGCACCGGCAATGTCACCCGCACGCCGCTCGCGGTAGCGAACCTCGACTTCGGCGTGATCAACCCGGGC<br>TGCCTCGGCCGCCGCAACCGCTACGGCTACTTCGGCGTGGGCGACCCCATGCCCAAGATCGGCGGGGTGGCCA<br>AGCTCGACTTCGACCGCGCCGGCCACGGCGACTGCACCGTGGCGCGCCGGGACTTCGGGCCGGGGTGCTTCGC<br>CGGGGAGCCGTTCTTCGTCCCCGACGACGTCGAGGGGAACGGCGACGAGGACGACGGCTACATGGTGTGCTAC<br>GTGCACGACGAGGGCACCGGCGACAACCGGTTCGTGGTGATGGACGCGCATTCGCCCGACCTGGACATCGTCG<br>CCGAGGTGATGCTTCCCTCCCGCGTCCCCTACGGCTTCCACGGCCTGTTCGTCACGCAGTCCGAGCTCCGGTCA |

| | | |
|---|---|---|
| | | CAGCACCAATGATCATCAGATTCAGACACACCAACACGTCCGATGTTTCCGCGCCATAGATTGCGCGCGACGAT<br>GTATAATAGTAGACCAACAGTGGTGCATGGGTAGGTCATGTATAGGATCCCATGACATACACATAGGAAAAATA<br>GGTCATCTCATGTGTAGCATTTACATGTTTACAAGAAAGAATTATTGGTGAAAAACGCATCTACAGTAAAATAAA<br>AACCTACCTCAG |
| *NCL1* | C2_transcript_<br>104852 | GATCAACTTTGAGGAGATTGACTTTGACAAGAATGACGCTGTCGATAGGATCATGGACGACTTTGATACTTCAGGCAA<br>TGACACTGTGGAGGAGGATGAGTTTGTCGCGGGAATGAAAATATGGCTTCATGAGGCGAATCAAAAATGGAGGCTAG<br>TGGTGCCTACTCTAATAAGTTTGTCAACGACTACCATGCTAGAACTAAGGAGGAGCATGACCAGTTGGTCGACAGACC<br>TGACGAGGCAGTGGAGAGCGTCGAGAACCCTGGCTGGTGCATTGCCAAAGCCGTGGGGTACCTGATTCTCGGTGCTA<br>CCATTTGTGCCGCATTTGCAGACCCGCTTGTTGATGCTGTCCACAACTTCTCTAACGCCACACACATCCCGTCCTTCTTT<br>GTCTCGTTCATCGGCCTCCCGCTGGCAACCAATTCCAGCGAGGCCGTCTCGGCCATCATCTTCGCCAGCAGGAAGAA<br>GCAGAGGACTTGCTCCCTCACTTTCTCAGAGGTATACGGCGGTGTGACCATGAACAACACGCTGTGCCTGGGCGTGTT<br>CTTGGCGCTCATCTACTTCAGGGAGCTGACGTGGGACTTCTCTTCGGAGGTGCTCGTCATCCTGCTCGTCTGTGTCATC<br>ATGGGCCTCTTCACAAGCTTCCGGACCAGCTTCCCGCTCTGGTCCTGCCTGGTGGCGTACCTGCTGTACCCTCTGACCC<br>TCGCCGTCGTCTACGTCCTCGACTTCGTCTTCGGCTGGTCATAGACCCATGCTCCTCCTCCTCGGCACACGCTTGAGTT<br>CGATCCTGTCATGATTTTTGACTCATTGTAATTTATCTGTCTAGATTTGGAGGCGTTTATTGTGTTCATATCC |
| *NCL2* | C2_transcript_<br>25511 | CTGAGGTCTAGGGTGGAGTGACCATGAACAACACGCTCTGCCTGGCAGTGTTCTTGGCTCTCGTCTACATCAGGGGCT<br>TAACTTGGGACTTCTCGTCAGAGGTCCTCGTCATCTTCCTCGTCTGCATCATAATGGGCCTCTTCACCAGCTTCCGGAC<br>CACGTTCCCGCTGTGGACGTGCTTCGTCGCATTCCTTCTGTACCCACTGTCGCTGATCTTGGTTTACATCCTCGACTACA<br>AGTTTGGCTGGTCGTAGACCTGAACCTGAACTGAATGGTGATTAGTTGATAGGTCTGATATACGGCATGGATCACTTCT<br>CTTACTGTTTGTAAACCTCATCTGGTACGTGTTTCCGGTTGGATGCAGACTATGGCTTTGACATATTGCATACTTGGAGC<br>CATCACTAATAAGTGAAGATGGAACTCTGTGATCATGATATCGCGTGTGTTGCTGCAGGAATTGGTTGCAGTATAGTTC<br>ATCTGAGATCTGTCATACGAAACATGAATCTATTGTATAGTCGTTAAACATCTGTTGACAAAC |
| *NHD1* | C2_transcript_<br>96748 | GAGAATCTGGAAGGCAGAGATTGAAAGTTCCACAAGCGCTTTCACGGATTGATACACAAGGAATTCTATTCTTCTTAG<br>GTATTCTGTTGTCGGTTGGCAGCTTGGAAGCTGCAGGGATTTTGAGGCAGTTGGCGAACTATCTTGATGCCAATATTCC<br>AAACGCTGACCTCATTGCAAGTATCATTGGTGTAGCATCAGCAATTATAGACAATGTTCCACTTGTTGCTGCAACCATG<br>GGGATGTATGACCTGACTTCATTTCCTCAAGATTCAGATTTCTGGCAGCTTGTTGCGTTCTGTGCTGGTACGGGTGGTTC |

| | | |
|---|---|---|
| | | GATGCTTATCATCGGGTCTGCAGCAGGAGTGGCTTTTATGGGAATGGAAAAGGTGGATTTCTTCTGGTACCTTCGCAAG<br>GTGAGCGGTTTCGCCCTTGCAGGCTATGTAGCTGGTATCATCACCTACCTAGCTGGTCAAAACATCCCTCTATCTCTTCC<br>CACTTCACTGGCGGAGATCCCGTTTATCGCGGGTTCGTGAAAGGCT |
| *NHX1* | C2_transcript_8989 | TGTGTGTTATGTCCTTTCAAGAATATACTCTTTTTACTTGCATGATGTGGGTTATTCAGATTATTATCGGTGGGTCTCATG<br>AGAGGTGCCGAGTCAATTGCATTGGCCTACAATAAGGTAAGGTATTGTGTTTGATCTATTCCATATGTGCTTTGGGTGTA<br>ATACATGATCAAGTTTGCTCTGAGTTATTGTTTTTGATGAAGTGCTGCTGTGACTTGCTTCATTTTGCCTTAACCTTTTTTT<br>CTCAAGAAACTAATAAGCATACTGACAGCTGACGAAGCTAAAAGGGAAAGAAACAGCCTTGTGTATGTTTTATTGTG<br>CTGCTGTGACCAGCTTCATTTTTCTTTAAGATTTTTTTTCCCAAGGAAGTACTAAGCATACTGACAGCTCACATGGAAA<br>AGGGAGGTCGCTGTCGCTGATAGATAACTTTGCAATTGTGAAGTTGAAATCAATGGTTCTTTAGTTGTTGCACAAACTG<br>TTGTCGCTGAGAACATAACTCTAGTTGGACAATGGTTGCCTTTGCTGCACTGTGCAAACTGACGCAGT |
| *NHX2* | C2_transcript_55688 | GGCTACTCGGAGGGGGGATGATGGGGTTGGGGCTGGGCGACCCGCCGGCGGATTACGGCTCCATCATGGCGGTGGGG<br>TTGTTCGTCGCGCTCATGTGCATCTGCATCATCGTCGGCCACCTCCTCGAGGAGAATCGCTGGATGAACGAGTCCACC<br>ACCGCGCTCTTGCTTGGGCTGGGCGCCGGCACGGTCATCCTCTTCGCGTCCAGCGGGAAGAACTCGCGCTTAATGGTC<br>TTCAGCGAGGATTTGTTCTTCATCTACCTGCTCCCACCCATCATTTTCAATGCAGGGTTCCAAGTGAAGAAGAAACAAT<br>TCTTCCGCAACTTCATGACTATTACATTGTTTGCTGTAGTTGGGACCTTGATCTCCTTCAGTATAATATCCCTTGGTGCGA<br>TGGGGCTAATATCAAGGCTTAACATAGGCTCGCTTGAGCTTGGAGACTACCTCGCACTTGGGGCAATATTCTCGGCAA<br>CGGACTCTGTTTGCACCTTGCAGGTGTTAAGCCAAGATGAGACACCCTTCTTGTACAGTTTGGTGTTTGGTGAAGGTGT<br>TGTTAACGATGCGACATCAGTTGTGTTGTTCAATGCAATCCAGAACTTTGATCTTGGGAATTTCAGTAGCCTCAAATTC<br>TTACAGTTCATTGGAAATTTCCTCTATCTATTCGGCGCCAGCACCTTTCTTGGAGTAGCTAGTGGACTTCTCAGTGCTTA<br>TGTCATCAAGAAACTGTACTTTGGCAGGCACTCCACTGATCGTGAAGATGCTATAATGATGCTCATGGCTTATTTATCTT<br>ACATGCTGGCTGAATTGCTTGATTTGAGTGGTATTCTCACGGTTTTCTTCTGTGGTATTGTAATGTCGCACTATACCTGGC<br>ACAATGTAACAGAGAGTTCCAGGGTCACAACCAAGCATGCCTTCGCCACGTTGTCATTCATCTCTGAGATGTTTCTCTT<br>TCTCTATGTTGGCATGGATGCATTGGATATAGAGAAGTGGAAGATTGTTAGTGAAACATATAGCCCAATGAAATCTATT<br>GCCTTGGGCTCCATTATTTTGGCGTTGGTGCTGGTTGCAAGAGCTGCATTTGTTTTCCCACTATCTTATCTCTCCAATTTG<br>ACCAAAAAAACTCCAGGCGAGAAGATCTCTATTAGGCAGCAAGTTATTATTTGGTGGGCGGGTCTCATGAGAGGTGC<br>CGAGTCAATTGCATTGGCCTACAATAAGTTTGCAAAATCAGGGCACACTCAGCTCCCTAGCAATGCTATCATGATCAC |

| | | |
|---|---|---|
| | | CAGTACAATCATTATTGTTCTTTTCAGCACAATTGTCTTTGGGCTACTGACTAAGCCATTGATCAGACCCCTGATTCCAG<br>CGAGGCACCTCACTAGGGAAGTGAGTGCCCTTTCTGAGCCATCCAGCCCAAAATCCTTCCTTGAACAGCTGACCGTG<br>AATGGGCCCGAGACAGATCTTGAGAACGGTGTGAGCATACGCCGCCCGACGAGCCTCCGGATGCTCCTGGCTAGTCC<br>AACACGGTCAGTCCACCACTACTGGCGCAAGTTTGACAACACCTTCATGCGGCCGATGTTCGGAGGGCGAGGCTTTG<br>TCCCATTTGTCCCTGGATCTCCCACCGAAAGCAGTCTACCATTACTAGCCCATGGAAGTGAGAACTAGAGGACTGCAC<br>AGAAGAGGAAGGGGAGGATCTGACAGGAAAAGAAGTTTTGTAAATTAGCTACAGTGAGTGTAATTGCTGTAAATCTG<br>AGGTAGAACGGTTCTTCGTAGCTGCTCATTTAGGCTAATTATCTGACCTGCATACAGTTTTTTTACCTGACCTGCATACA<br>GCTGTGTATATCTCACTAGCCACTGCACATTATAAAGGAGTATTCGCCCT |
| *NHX6* | C2_transcript_<br>42547 | CATCATTAGCAGAAACCTTTGTGTAAGTCAACATTAAAAAGTTTTATGTTTTGGTAAATTTACATACATCCTTGTCTATTC<br>TGTTCTCCCTACAGGTTCATATATATGGGTTTTGATATTGCGATGGAGCGTCAAAGCTGGTCACACATTGGGTTCATATT<br>TTTCTCAGTTATCTTCATATTAGTTGCAAGGGCTGCAAATGTTTTCTCGTGTGCTTTCTTGGTAAATCTGGTACGGCCACC<br>TCACCGGCAAATATCCAGGCAATATCAACAGGCACTTTGGTATAGTGGTCTTAGAGGGGCTATGGCTTTTGCCCTTGCT<br>CTTCAATCTGTTCATGATCTTCCTGACGGCCATGGACAGACAATTTTGAGTGCTACCATATCTATAGTTGTTCTAACAGT<br>ACTTCTGATTGGAAGCTCAACAAGCACAATGCTTGAAGCTCTACATGTTGTGGGAGATGGTAATG |
| *NHX7.1* | C2_transcript_<br>51996 | CTCTCTGTCTGTTAAACGTGCTAGTGATTCAGTTCAACCTTATCTGAAATCAGAAGTCGGAACAATGTTTGTGTTCTTC<br>ACAGGTGGTATCGTGTTTCTGACATTGATTTTGAATGGTTCTACCACACAATTTTTGTTGCACCTGCTTGGCTTGGGAAA<br>ATTGTCAGCAACAAAGCTTCGTGTATTGAAGTATACACGGTATGAAATGCTAAACAAGGCATTGGAGGCTTTTGGTGA<br>TCTCAGGGATGATGAGGACCTTGGGCCTGCTGATTGGATTACTGTGAAGAAACATATCACATGTCTGAATAACTTGGA<br>AGATGAACAAGCACATCCCCATGATGTTCCTGACAAGGATGATCACGTACATACCATGAATTTGGAGGATACTCGAGT<br>GCGACTTTTGAATGGTATGGAATATACATGATAA |
| *NHX7.2* | C2_transcript_<br>1851 | GATTTATTTTCAATGATACAATCATAGAGATTTCGCTTACCCTTGCTGTCAGCTATATTGCTTTCTTCACTGTAAGATAAT<br>CAGATAACAAACATATATATCAAAGCTCCCATATTTCTGTCTTTTTTTTGTCTAGAAGGACATGATTCACTTAATGTTTTG<br>AAATAGTTCATTTCCTGGAATAATTTGTCGTTTCAGTCATAGAATGATGGTCAAGCAGCCTTTTTGCAACATTGGGATGC<br>TTTTTTTACTGGTAAACTGAAATGACTTGTAGCTTCTAGATATGCACATAGCAAAGTACCACATGATTTTGAAGTCGCA<br>GAGCTCGACAGGGCTTCATGAATGTCCTCAGACATAATTATGTGGTTTAAACAAACATTCCGGTTTGAGGGTATATTAG<br>AGGATTCATGTTGGAACCAAAATTCGATTACTATTTGTACTTCTGACTCTAACTTCAACTAACTTGAGTGATGTTTGGTG |

| | | |
|---|---|---|
| | | AGCTAACTGAATTTATGTCATGAGCACGGACATTGCCTTTTTTCTAGTAAAAATCTTGAGGAAAACAGGATTCTAGATT<br>TTTGATATAGCAAGAAATGGTTTGTTTGCCATTTTAAGCTAGTCCAATCATTTTACATTCTTTCCAGCTTTTGGACATGTT<br>GACTGCGTACCATTTAACTGATGTTAATCCATATGAGGTGTCAATTTTGTTGAAGTTAATAAATATAAGTATTCATTGGTT<br>GTAGCTTGACATTGCTTGTGCATGATTAATCTGTTCTGTCATGTCTGCTGCAAGACAAGTTTCAGCCATAAGTCTGAATA<br>TAGACTTACCATTCAAGATGTGACACTGCCCAGTCCTTCAGTTTTTTGGGGGCTGTTTTCTATTACTAATATTGTTTATAT<br>CAGGCGCAAGATGCATTGGAGATCTTTGGTGTTTTGGCCGTCATGACCTTGGGGATGTTCTATGCTGCTTTCGCAAAAA<br>CTGCTTTTAAGGGTGACAGCCAGGAAAGTTTGCATCATTTCTGGGAAATGGTGGCTTACATTGCGAACACACTTATTTT<br>CATACTGAGTGGGGTTGTTATTGCAGATGGTGTACTGCAAGATAATATTCATTTTGAGAGGCATGGCACATCATGGGGG<br>TTTCTTCTTCTGCTCTATGTGTTTGTGCAAATATCGCGTGCTGTAGTTGTCAGTGTTTTGTATCCATTATTGAGTCACTTTG<br>GATATGGTATGGACGTCAAAGAAGCCACAGTTCTTGTTTGGTCAGGGCTGCGAGGGGCCGTTGCTCTATCACTCTCTCT<br>GTCTGTTAAAG |
| *NHX8* | C2_transcript_<br>26994 | CGGCGGAGTCACCCACACTCCCTCCGCTCTCGCCCCCACAGCCGCACGACCGGCGGCCTCGTCTCCCACCGCCGCCG<br>CCGCCTCTCCCTCGCACGCCTCGCCGGCACCTCTCCCTCACCCGCGTCACTGGCGCCTCTCCCTCTCGGGCCGTTGTTG<br>CCTCTCCGTTGGCCCCGTCATTGTCGCCCCTGATGTGCTGCGGCGCCGTGACCCGGCCTCACAGGCACAGCCAGGGTG<br>ACAACAGCTCCCCGACGCCGCCGCCCTCCGCAAGGAGCGACGACAGCTCGAAGCTCTGCGGCACGGCGTGCGCTGA<br>GCGACGCGCCGCGTGGGCACCGCCGCCTCAGCCACCGGCGTGGCCGACCACCCGTCCACGTCCCCATCGCTGCCGC<br>CGGCCGACCACTCGACGTCACGCTCGTCCAGCTCGAAGCACGCGCCGTCGTGGCCCGCCTCCGGCTGCTTCAGCAGG<br>CCAAGAAACCGAAACGCCCCCCGCGCCCGGCGACCTCGGCATCTCCATCGATCGTCTCAGCAAGCGCTACCTCGCCC<br>GGCTCCCGCATTGCTGATGCAAGCCAAGAAACAGGGAGTGCCGGCAGTAGTACTCTGAATTCATCAATGCAACTGAA<br>TCCAAACATGTGAATCAAGTTGTGGCAGTTTGTGTTCTTCACTGGTGGCATCGTGTTTCTGACACTGATATTGAATGGTT<br>CTACCACACAATTTTTGCTGCACGCACTTGGTATGGACAACCTGTCAGTAACAAAGGTGTGCAAGCCAGTTACTGGGC<br>AATGCTTGACGAGGGACGGATAAATGCAGCTACAGCCAATATTTTGATGCGATCAGTTGATGAAGCTATGTATCTTGTT<br>TCCCATTATGCGATTGGAACAGTTTGCAGTCCAGTGTCCATTTCCCAAGTTACTACAGGTTCCTTTCTGCAAGCATGTTA<br>CCACAAAGTTTTATCACATAATTCACAGTACAAATATTGGAGTCAGGATGTTACATCTGTGCTGCATTTCTTCATGCTCA<br>TAGAATTGCAAGGAGATTTTACCAATATTGGAAAACTTTTAGAATGCTTTTATGTGCTTGTTATTTACCACTTCTTTCCTA<br>CCAATTCCAGAACTTAGTATCCAATCCATGGATCGGAGGGAGTAAATGAATATTAGTCACACTACGTGGCGCACATGA |

| | | |
|---|---|---|
| | | CAGATATTATGTGTAGGTGTCCGTCGTTATAGTCCATAAGCAGAACAGAAAGTAGTATTTTGTTACTTGTCGGAGCCAG<br>CTACAGACCCTATTTTTTAAGATACGTGGAAAGTAGTATGGTGCAGATGGACGCATTGCTCAAACCATTTAGCTTTTTA<br>AGGTTGAACTATGTAAAGCCTGTCTAAAGTCATCGTCATTTGTGATGTCCTGTTCTAGTATCACCCCTTCAGAGGGACT<br>ATTATGCAATACTGACATGGTAGGTTCTTTTGGTTTGTTGTTTTTGTCCAAATCTGTTAGGCACGATTGATGAAATGATC<br>AAATAAAAACCATTTGATAATGCATGTTATTTACAAAAGCCCCATACCTGGAGTCATTGGGTAATACTCGTCAATGTTG<br>TGACTCGGTGCGGGAACGATTTCTGGGATGGATTCGTTGACATTTATACCCTCATTATGGCCTGTAACACGGAAGTTAT<br>ATGCAATGTTGTGTTTATACCAGAATGAAGGTCAACAAGACGGGGTAGAGGTATACCTGGACTTCCTTGGGGATGTAG<br>ATCAACAAGAGGTGGTAGAGGTCAACATGAGAAGATCAAATAAGAACTACTCCCTCCGTTCCAAAATAGATGACTCA<br>ACTTTATACTAACTTTAGTACAAAGTTAGTACAAAGTTGGATCATCTATTTTGGAACGGAGGGAGTATTTGATAATGCA<br>CGTTATTTACAAAAGCCCCATACCTGGAGTAAT |
| *POD* | C2_transcript<br>_95233 | GTGTACGTCTGCGTCCGCATATATTTGTGGGGACGGCGAACACGCGCTATCTACCGAGCTAGGACGGCGGGCGACATG<br>AGTCCGGCCGTGGCGGCGCTCCTGGCGGTGGCGGTGCTGGCCGCGAGCGCCAACGTGTGCGCGGCGCAGCTCCGGC<br>GGGACCATTACGCCGGCGTGTGCCCCGACGTGGAAGCCATCGTCCGGGGCGCCGTCGCCAAGAAGTTCCAGCAGAC<br>CTTCATCACCGTCGGCGCCACCGTGCACGTCTTCTTCCACGACTGCTTCGTCGAGGGGTGCGACGCGTCGGTGCTGAT<br>CGCGTCCACGGCCAACAACACGGCGGAGAAGGACAGCACTGCCAACCTGTCCCTGGCCGGCAACGGCTTCGACAC<br>GGTGATCAAGGCCAAGGCGGCCGTCGACGCCGTGCCACGGTGCCGGAACCGGGTCTCCTGCGCCGACATACTTGTCA<br>TGGCCACCAGGGACGCCATTGCACTGGCCGCCGGGCCGTCGTACGTGGTGGAGCTGGGGCGGCTGGACGGGCGGAG<br>CTCGACGGCGAGCAGCGTCCCCGGCAAGCTGGCCCCGCCAACGTCCAGCCTCGATCAGCTCACGGCGCTCTTCGCCA<br>CCAACGGGCTCTCGCAGACCGACATGATCGCCCTCTCCGTGCATGAAGGAGGGCACACGGTGGGGCTGGCTCACTGC<br>AAGACGTTCGCCGGGCGGCTGCGGCCGACAGCCGACCCGACGCTGAGCCCGCGGTTCGCAGCCCAACTGCAGGCGT<br>GGTGCCCACCCAACGTGGACCCACGGACCGCCGTGCCCATGGACACAGTGACGCCGCGGGCATTTGACAACCAGTA<br>CTTCAAGAATCTTCAAGGGGGGATGGGCCTACTGAGCTCCGACCAGCTGCTGTTCACCGACCCGAGGTCCAGGCCCA<br>CCGTCGACGCGTGGGCCCGGAGCGGTGCCGCGATCGACCGGGCCTTCGTGGTGGCCATAACAAAGCTGGGCCGCGT<br>GGGGGTCAAGACCGATGCGTCCCAGGGGAACATACGCCACAACTGTGCAGCGTTCAACTGAATTCAACTGCTATTGC<br>TCTGCCATTGTCCACAAAGGAATGAGAAATACCAGTAGTATATACGTATCAAAATTGTAGTTCAAGGGAAATGTATCCT<br>AATTGTTTGTGATGGTGCCCGTTCAGTTGGCAATCGACAGGTTGTTCTTATATTGATTCAGTTTGGGATATGAAAGTTTC |

| | | |
|---|---|---|
| | | AGTCAACAGAGAGGAGAAGGTTGTTTGCACAAACTTGGTGCTACGGCTCGTCGTGTGCCTCGCCAAAATAAAGAAGTACAATTGACTATCGTATT |
| *PSA1* | C2_transcript_29369 | TGAAAAAGAAAATAATAAAAAAGGTTGTGAAAGTTGTTCGAAAAAAGCCAACTGCTGAAGCTCCAGTGGATAAATCGCCTCAGGTTGACAAGAATGCCGTGGCAGAAACTGCGAGCAAAACCGTCGAAAAGCACATTGAACAGAAAAGTGAGGATCTTGGGAAAGAGAAGGCAGGGAGCTGGCATCGTTCAACAGCCTGAGGCTAAGAAAACTGGGAAGAAGAAGGTCATTCGAAGGATTGTTAAAAGAAAAGTTCCTGCTTCAGCGACTGAGCCAACAGCCCTTGCTGCACCTGCTGAAGCAACTAAACAAGATGTGGATGTTCAGCCGGAGAAGATTGTTGAAGGCGTCACTGATGCTGGGAATTCACAGACTAAGCTGGAGGAAGGATTGAAAACTCCTGCTGAAGATGTTTCAAACCAGAAGAAAGAAGAAGGATTGAAAACTCCTGCTGAAGATATTTCAAACCAGAAGAAAGAAGAAGGATTGAAAACTCCTGCTGAAGATACCTCAAATCAGAAGAAAGAACAGGAGCTGGAGATAAAGGGAGACATAATGACTGATGATCAAAAGGCAAATACAGATAAGGTTAACCAGCAGGAAGTTGTGGAACAGAAAGATCCGAAAATTGATGAAACAAACGGAAAGAGTGACAAGAAAAAGGATGACAACGAAACAAAGGATAAAGACCAGAAGATGGACTCAAAGAAAAAGTCGCCCATTGACACCAAAGAAAAGAAGA |

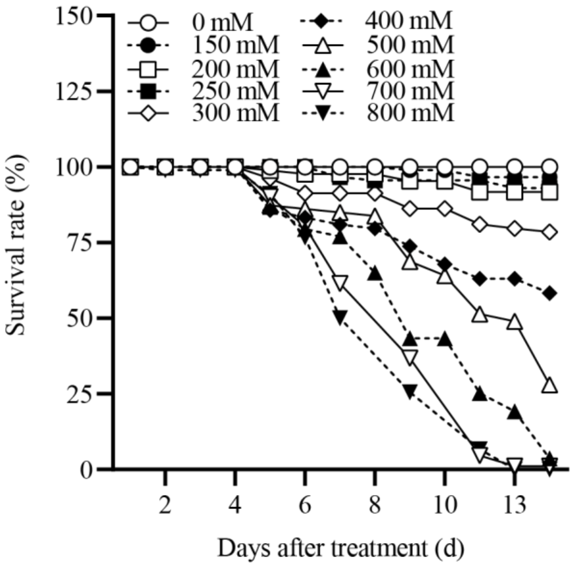


**Figure S1.** Dynamic response of survival rate of tall wheatgrass line Zhongyan 1 to different concentration of NaCl.

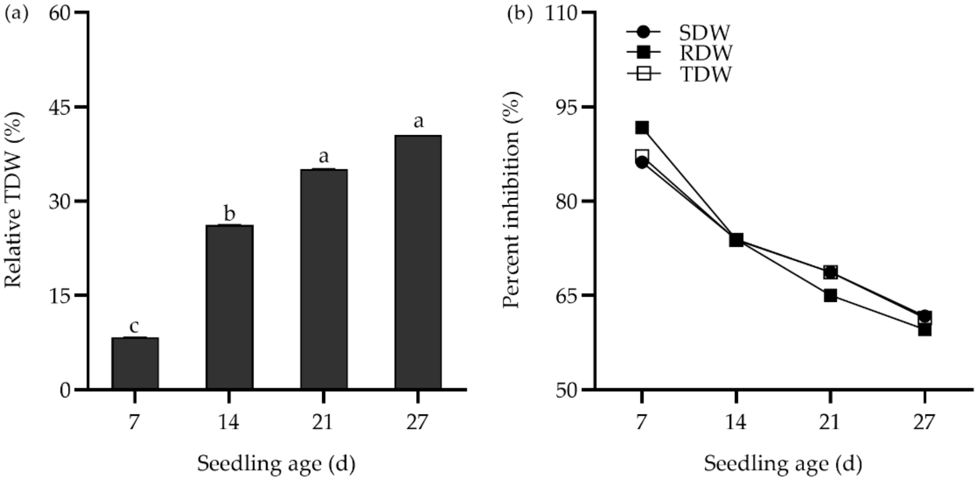


**Figure S2**. Comparison of salt tolerance in tall wheatgrass line Zhongyan 1 seedlings at different age. (**a**) Relative total dry weight per plant (TDW) to the non-stress control; (**b**) percent inhibition of dry weight per plant. Data are represented as mean ± SE (n=20). Different letters indicate that the difference was significant at $p < 0.05$.

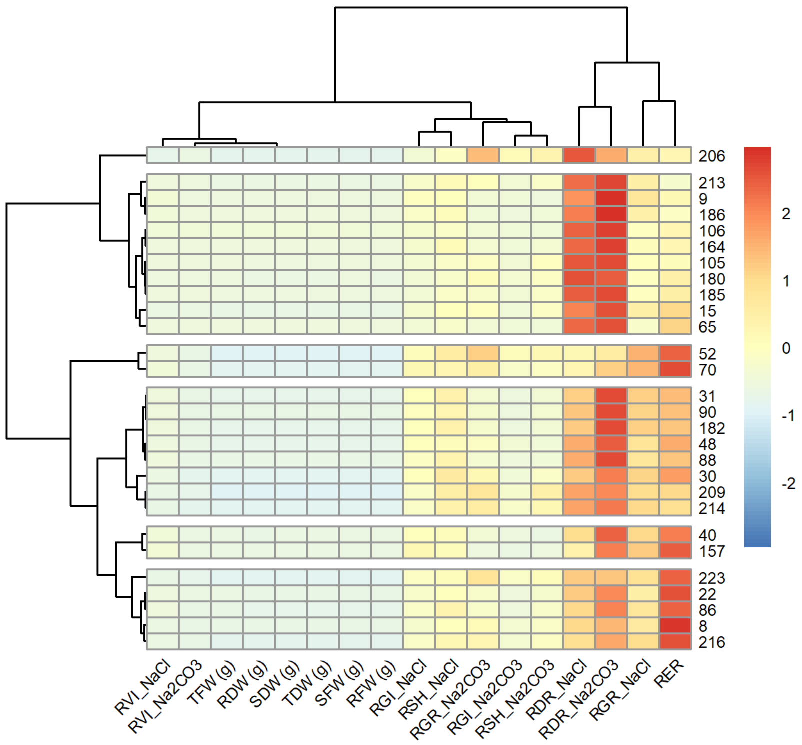


**Figure S3**. Hierarchical cluster analysis of 28 tall wheatgrass lines according to salt tolerance combining seed germination rate, seedling emergence rate, and seedling growth traits. RGR, relative seed germination rate; RDR, relative salt damage rate; RGI, relative seed germination index; RSH, relative shoot height; RVI, relative vigor index; RER, relative seedling emergence rate; SL, the number of senescent leaves; GL, the number of green leaves; SFW, shoot fresh weight; RFW, root fresh weight; TFW, total fresh weight; SDW, shoot dry weight; RDW, root dry weight; TDW, total dry weight; R/S, the ratio of root to shoot dry weight. NaCl, 250 mM NaCl; _Na2CO3, 100 mM $Na_2CO_3$.

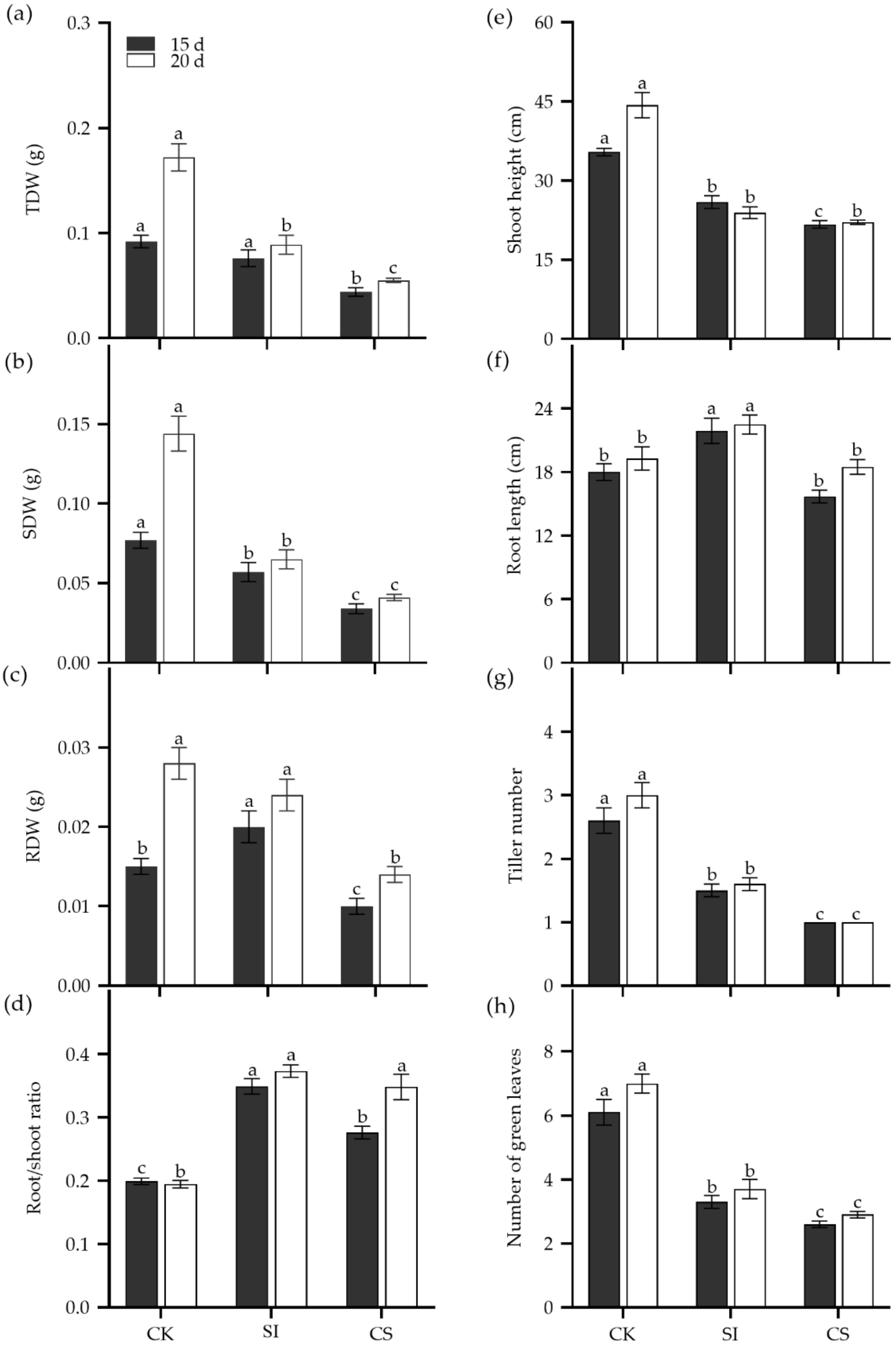


**Figure S4.** Comparison of the effects of non-stress control (CK) and salt stress resulting from stepwise increase (SI) and sudden increase (CS) to 300 mM NaCl on seedling growth of tall wheatgrass line Zhongyan 1. (**a**) Total dry weight (TDW); (**b**) shoot dry weight (SDW); (**c**) root dry weight (RDW); (**d**) root/Shoot ratio; (**e**) shoot height; (**f**) root length; (**g**) tiller number; (**h**) the number of green leaves. Data are represented as mean ± SE (n=10). Different letters indicated that the difference was significant at $p < 0.05$.
